# Core components of physical activity interventions in the prevention and management of breast cancer-related lymphoedema (BCRL): A scoping review

**DOI:** 10.1371/journal.pone.0355189

**Published:** 2026-09-02

**Authors:** Laura Henry, Julie S. Wilson, Jason J. Wilson, Esther R. Beck

**Affiliations:** 1 Institute of Nursing and Health Research, School of Health Sciences, Ulster University, Belfast, United Kingdom; 2 School of Nursing and Paramedic Science, Ulster University, Belfast, United Kingdom; 3 School of Sport and Exercise Science, Ulster University, Derry/Londonderry, United Kingdom; Chinese University of Hong Kong, HONG KONG

## Abstract

**Background:**

Breast cancer-related lymphoedema, a chronic and debilitating swelling condition, can occur as a sequela of breast cancer treatment. Physical activity is a key recommendation of cancer survivorship guidelines. However, uncertainties that remain around physical activity and breast cancer-related lymphoedema management, have potentially contributed to low physical activity levels in this population.

**Aim:**

The aim of this scoping review was to explore the core components of physical activity interventions to prevent or manage breast cancer-related lymphoedema and to understand the context in which they are developed and delivered.

**Methods:**

This scoping review was conducted in accordance with the Joanna Brigg’s Institute methodology. A systematic search of the literature was conducted across five databases (Medline, CINAHL, Embase, SportsDiscus, Scopus) including grey literature. Primary research studies with physical activity, both structured and unstructured, as the main intervention and an outcome for breast cancer-related lymphoedema were included.

**Results:**

After full title and abstract screening, 79 studies were charted. The majority were randomised controlled trials (n = 44; 55.7%), incorporated resistance exercise (n = 43; 54.4%), typically lasted 12 weeks (n = 21; 26.6%), and were supervised (n = 34; 43%). Participants included were most frequently diagnosed with stage II breast cancer (n = 17; 21.5%) and had received the breast cancer treatments axillary node surgery (n = 64; 81%) and/or radiotherapy (n = 55; 69.6%). Lymphoedema was most frequently measured by arm circumference (n = 43; 54.4%). Motivational strategies (n = 27; 34.2%) or behavioural change theories (n = 5; 6.3%) were included to encourage physical activity participation.

**Conclusions:**

Physical activity interventions focused on short-term lymphoedema goals at the expense of encouraging long-term physical activity behaviour. To improve physical activity levels in this population, five future research recommendations have been proposed: recognise the physical activity preferences of this population; explore the impact of lymphoedema risk factors; target research early in the cancer timeline; apply behavioural change theories; explore lymphoedema outcomes.

## Introduction

### Rationale

Breast cancer occurs in 1 in 7 women in the United Kingdom [1]. The common breast cancer treatments, namely axillary lymph node dissection (ALND) and regional lymph node radiation (RLNR), can damage the lymphatics resulting in the chronic and debilitating condition breast cancer-related lymphoedema (BCRL) [2]. BCRL typically presents as swelling of the upper limb, chest wall or trunk and may be associated with heaviness, stiffness, numbness, tightness, fatigue, weakness and decreased coordination and mobility in the affected arm [3]. Research reports that up to 30.1% of breast cancer survivors (BCS) who receive both ALND and RLNR will experience BCRL within five years of treatment [2]. However, with no internationally agreed definition of BCRL, nor a gold standard measuring tool, determining who will develop BCRL and confirming a diagnosis can be difficult [4].

Physical activity (PA) commonly defined as “any bodily movement…..that requires energy expenditure” [5] is one of the key recommendations in cancer survivorship guidelines [6] as it improves quality of life (QOL), reduces anxiety [7]; reduces cancer reoccurrence and mortality [8]; and reduces treatment–related side effects such as fatigue and depression [9]. However, for BCS, the historical anecdotal assumption that over-exertion of the affected limb could fatigue or overload the lymphatics, resulting in swelling [10], may have contributed to PA caution amongst healthcare professionals and patients alike [11]. Interestingly, self-reported PA levels of BCS with BCRL, measured via the International Physical Activity Questionnaire- Short Form (IPAQ-SF), have been reported as significantly lower (p = 0.046), when compared to healthy controls [12]. Whilst recent 2025 United Kingdon (UK) breast cancer lymphoedema guidance [13] now advises that PA is safe and may improve QOL for those at risk of, or living with BCRL, they do not provide tailored PA guidance to this population but refer to previous generic PA guidance for healthy adults [14,15].

To further understand the gaps within the literature surrounding PA and BCRL, a preliminary search of MEDLINE, CINAHL, PROSPERO and Open Science Framework was conducted between September 2024 and November 2024. Previous systematic reviews and meta-analyses on this topic have focused on evaluating the safety and efficacy of PA or exercise interventions on lymphoedema outcomes [16–28]. Others have tried to determine the optimal exercise dosage to reduce or prevent BCRL [29], or determine the need for those with BCRL to wear compression armsleeves [30]. However, in this preliminary search there were no systematic or scoping reviews that considered the core components of PA interventions for those at risk of, or living with, BCRL.

Therefore, it is the aim of this scoping review to explore the core components of PA interventions to prevent or manage BCRL and to understand the context in which they are developed and delivered. This will be achieved through five objectives:

1. What are the characteristics of the included participants (e.g., age, BMI, cancer stage)?
2. What are the characteristics of the PA interventions, e.g., type, frequency, duration, intensity?
3. What outcomes are measured?
4. What are the participants’ volumes and patterns of PA at baseline, during and post PA intervention?
5. What are the knowledge gaps and areas for further research?

## Methods

A systematic scoping review conducted in accordance with the Joanna Brigg’s Institute (JBI) methodology for conducting scoping reviews [31] was chosen to explore and synthesise the literature regarding PA and BCRL. A scoping review which aims to map multiple types of evidence, was felt most appropriate in order to identify gaps and priorities for further research [32]. This approach was reported in line with the PRISMA-ScR (Preferred Reporting Items for Systematic reviews and Meta-Analysis extension for Scoping Reviews) checklist (S1 Table) [33]. The web-based software programme Covidence was used to streamline the data extraction process [34,35].

### Protocol and Registration

The scoping review protocol was registered with Open Science Framework on 25-11-24, updated on 22-01-25 and can be found at: https://doi.org/10.17605/OSF.IO/T6CUQ.

### Inclusion Criteria

Eligibility criteria were informed by the participant, concept, context (PCC) questions development framework.

### Participant

Studies that included: 1) ≥18 year old adult participants; 2) who had treatment for breast cancer which included, removal of one or more lymph nodes and/or radiotherapy to the chest or axilla and therefore were considered at risk of BCRL; 3) or those who had a formal diagnosis of BCRL by a healthcare professional. Participants with active malignant disease, or acute oedema were excluded.

### Concept

Studies that include all types of PA, both structured and unstructured, as the main primary intervention and stated a recognised outcome measure for lymphoedema.

### Context

Any environment or setting (clinical, home, community, online).

### Search Strategy

Two concepts defined the search strategy, 1) Physical Activity. 2) Breast Cancer Lymphoedema. A combination of free-text term and Medical Subject Heading (MeSH) were used where available. Truncation (* or #) was used to account for different spellings of similar terms and Boolean terms (AND, OR) was used to combine different search terms. Results were searched from the year 2000, the year when there was a shift in research regarding PA as a contraindication for BCRL (35), to the current year and were limited to primary research studies in the English language where full text was available. The search strategy was initially developed and piloted in two databases, Medline and CINAHL to identify any further keywords and index terms in the title and abstract of retrieved papers. A Subject Librarian (KC) was consulted throughout this process. Once the final search strategy was defined and completed in Medline and CINAHL, it was translated onto a further three databases Embase, SportsDiscus and Scopus.

Grey literature was extensively searched across a list of relevant home pages for key societies and professional organisations (S4 Table). This list was compiled by the main author (LH), a Clinical Specialist in Lymphoedema and Oncology and a second independent Lymphoedema and Oncology expert (DW) was consulted. Targeted website searching via Google Scholar was also carried out, to locate webpages and/or documents published on the internet. The grey literature databases of PROSPERO, TRIP, OpenGrey, Web of Science and Scopus Proquest Conference Papers Index were searched. Institutional repositories and journals were sought from CORE, and any relevant completed clinical trials were sought from Clinical trials.gov. Reference lists of retrieved full-text articles were screened for any additional content. The search was then rerun on 11^th^ June 2025 before data charting was completed. The search strategy from the database Medline is presented in Table 1 and the search strategy from the database CINAHL is presented in S5 Table.

**Table 1:** Ovid Medline search strategy.

#	Searches
1.	exp sport/
2.	exp exercise/
3.	(“Physical Activ*” or PA or Activ* or Exercis* or Housework or ADL* or Recreation or Danc* or Garden* or Aerobic* or Fitness or “Physical Endurance” or “Activ* Energy Expenditure*” or “Caloric Expenditure*” or “Human Physical Conditioning” or “Circuit Train*” or “Circuit-Based Train*” or “Circuit Based Train*” or Plyometric or Stretch* or Drill* or “Strength Train*” or “Resist* Train*” or “Weight* Lift*” or Weight-Lift* or Weightlift* or Bicycl* or Bike or Biking or Cycl* or Walk* or Ambulat* or Run* or Jog* or Stand* or “Aqua* Therapy” or Swim* or Row* or Yoga or “Tai Chi” or Pilates or Inactiv* or “Sedent* behavio?r*” or “Sedent* lifestyle*” or “Stationary behavio?r*” or “Screen time” or “Health behavio?r” or “Health Promotion” or “Body Weight Change*” or “Weight Reduction Program*” or “Weight Gain Prevention” or “Body weight maintenance” or Sport*).mp. [mp = title, book title, abstract, original title, name of substance word, subject heading word, floating sub-heading word, keyword heading word, organism supplementary concept word, protocol supplementary concept word, rare disease supplementary concept word, unique identifier, synonyms, population supplementary concept word, anatomy supplementary concept word]
4.	1 or 2 or 3
5.	Breast Cancer Lymphedema/
6.	(“Breast cancer* lymph?edema” or “Breast neoplasm* lymph?edema” or “breast carcinoma* lymph?edema” or “breast tum?ur* lymph?dema” or “breast malignanc* lymph?edema” or “Postmastectomy lymph?edema” or “post-mastectomy lymph?edema” or “post mastectomy lymph?edema” or “breast cancer related lymph?edema” or “breast cancer-related lymph?edema” or “breast cancer-treatment lymph?edema” or “breast cancer treatment lymph?edema” or BCRL).mp. [mp = title, book title, abstract, original title, name of substance word, subject heading word, floating sub-heading word, keyword heading word, organism supplementary concept word, protocol supplementary concept word, rare disease supplementary concept word, unique identifier, synonyms, population supplementary concept word, anatomy supplementary concept word]
7.	5 or 6
8.	4 and 7
9.	limit 8 to (yr=“2000 -Current” and english)

### Study selection

All relevant results were imported onto the software management tool, Covidence, for removal of duplications and screening [34]. Two reviewers (LH and JSW) independently pilot screened the title and abstract of the first 25 articles, ordered by their Covidence ID, i.e., the ID assigned to them by the software Covidence. Once 75% agreement was reached, further title and abstract screening was completed. These two reviewers also conducted full text article screening, according to eligibility criteria. Any disagreements were resolved by discussion with the third and fourth reviewers (JJW and ERB).

### Data Charting

A data extraction table was developed in agreement with the research team to systematically collect and organise the data. It was initially piloted by two authors (LH and JSW) and further modifications were made in consultation with the full research team. From the initial review of the included studies, it was identified that most of the included interventions were planned, structured and repetitive, thus resembling exercise which is defined as a subset of PA [36]. Therefore to better consolidate the data from the reported interventions, the data chart was adapted to include the 16 items, internationally endorsed, Consensus on Exercise Reporting Template (CERT) [37]. This template was designed to aid replication of exercise prescription for research reports [38]. The inclusion of CERT in this scoping review was not to evaluate the included interventions but to identify gaps in reporting [37].

How PA intensity was reported varied within the studies. Therefore to aid charting of this data, the American College of Sports Medicine (ACSM) approximate classification of exercise intensity was consulted [39]. This chart was developed based upon commonly used methods of reporting intensity in practice such as %HRmax (percent of maximal heart rate) or %1RM (percentage of 1 repetition maximum). Other assumptions and simplifications made to assist with data charting included: 1) The timepoint to when participants were recruited to the interventions was categorised according to the weeks/months since breast cancer diagnosis or active breast cancer treatment. 2) Outcomes of interest reported, other than lymphoedema outcomes, focused on QOL, pain, strength, fatigue, function/disabilities, range of motion (ROM), PA levels, cardiovascular function, perceived exertion, intervention feedback or qualitative interviews.

All data chartered was in alignment with the study objectives to ensure the following data was captured:

i. Participant characteristics
ii. PA intervention characteristics
iii. Outcome measures
iv. Participants’ PA behaviours

Where data was missing or when additional data was required, the authors of five papers were contacted. Two authors responded and clearly answered the questions [40,41], two authors were not contactable but the data from their studies was included in the scoping review as the authors agreed that comprehensive data charting could still take place [42,43]. The final author contacted did not respond to email contact and as data from this study could not be comprehensively charted, it was excluded from the final number of studies for review.

## Results

**Fig 1:**
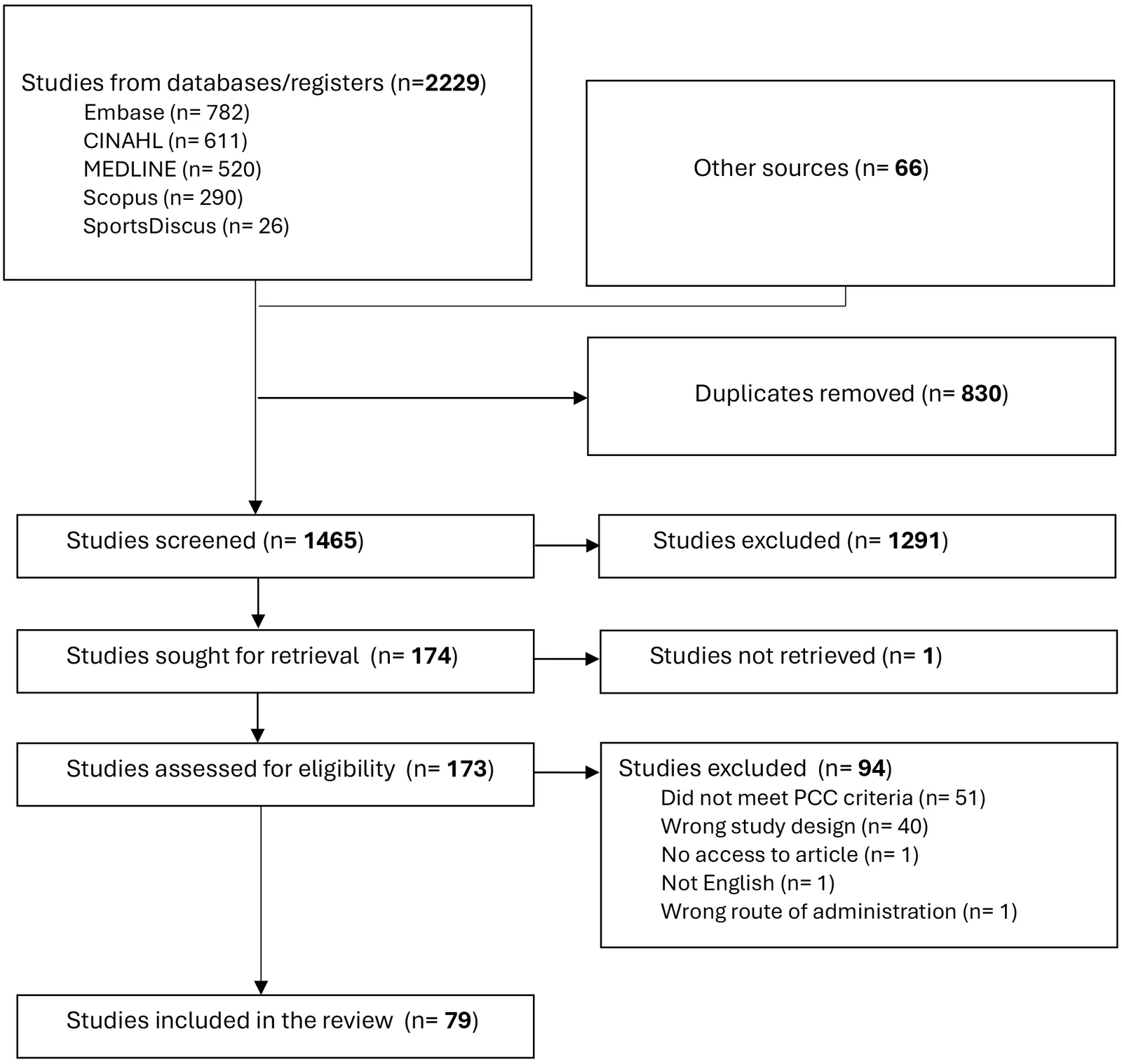
PRISMA flow diagram for selection of studies. Adapted from Covidence systematic review software, Veritas Health Innovation, Melbourne, Australia. Available at www.covidence.org [34].

The initial literature search across the five databases and grey literature led to 2,277 records. A further search of the reference list of full-text articles was completed, and the search strategy was rerun across all databases on 11^th^ June 2025. This identified a further 18 records, resulting in a total of 2295 records, 830 of which were removed due to duplication (Fig 1). The title and abstract of 1465 records were subsequently screened according to eligibility criteria. This resulted in full text screening of 173 records with a total of 79 finally included [40–118]. Main reasons for non-inclusion at this stage were: did not meet the PCC criteria as defined above (n = 51) and wrong study design (n = 40). Unless noted, the total number of studies (n = 79) was used as the denominator to calculate percentages.

The 79 studies included in this scoping review came from 26 worldwide countries and were published between 2003 and 2025 (S6 Table), with 50.6% (n = 40) published since 2017 highlighting the popularity this topic has gained in recent years. The most common study type, RCT, accounted for 55.7% (n = 44) of the studies. Other study types included: single group 19% (n = 15), randomised crossover 10.1% (n = 8), non-RCT 10.1% (n = 8), cohort 2.5% (n = 2), case report 1.3% (n = 1) and longitudinal follow up of RCT 1.3% (n = 1).

### Participant characteristics

Sample sizes of the interventions spanned a wide range, from n = 3 [113] to n = 1000 [117]. Participants included in the interventions as shown in S6 Table, were most frequently (n = 46; 58.2%) 50–60 years (mean or median age of the total sample, or the total of all groups), diagnosed with stage II breast cancer (n = 17; 21.5%) (S1 Fig.) [41,42,44,48,50,58,63,83,88,90,96,97,102,106,108,111,114], were less than 6 months post diagnosis or surgery (n = 21; 26.6%) or in contrast beyond 60 months post active treatment, surgery or diagnosis (n = 21; 26.6%) and were classified as overweight or obese (where BMI was reported within national obesity guidance categories [119]). There was only one male participant reported in one study [54].

Participants received a range of breast cancer treatments (S2 Fig.). Considering the risk factors associated with BCRL, it is not surprising that the majority of studies reported participants received surgery to the axillary nodes (n = 64; 81.0%) [40,43–61,63,65,67,71,73–76,78,79,82–92,94–96,98–106,108–118], radiotherapy (n = 55; 69.6%) [41,44–47,51,52,54–62,66,69,71,73–77,79–87,89,90,92,94–96,98,100,102–106,109–116,118] and chemotherapy (n = 54; 68.4%) [41,43–47,50–54,56–62,64,69,71,73–77,82–85,87–90,92,94–96,98,100–102,104–106,109–116,118].

### Physical Activity Intervention Characteristics

With the inclusion of all types of PA in this scoping review, both structured and unstructured, the results demonstrated a wide variety of PA interventions. The majority of the studies (n = 43; 54.4%) incorporated some form of resistance exercise (RE) which focused primarily on strengthening of the upper body (n = 20; 25.3%), with a few (n = 11; 13.9%) considering both upper and lower body strengthening (S6 Table). 15.2% (n = 12) of the interventions resembled PA recommendations found in a PA guideline [120] by incorporating both a detailed RE and aerobic exercise (AE) element (S6 Table).

Other types of PA interventions ranged from those incorporating gentle arm exercises to improve shoulder ROM and/or lymphatic drainage (n = 11; 13.9%) [55,58,65,72,73,76,91,94,96,114,117] to those with a holistic, whole-body approach (n = 25; 31.6%) (S6 Table) such as: aquatic therapy (n = 7; 8.9%) [41,45,63,79,87,112,113]; Yoga (n = 6; 7.6%) [66,68,89,92,100,116]; Nordic or pole walking (n = 4; 5.1%) [42,80,81,90]; dragon boat racing (n = 2; 2.5%) [54,85]; Pilates (n = 2; 2.5%) [107,111]; aquatic therapy versus Pilates (n = 1; 1.3%) [97]; football (n = 1; 1.3%) [52]; Qigong (n = 1; 1.3%) [69]; virtual reality games (n = 1; 1.3%) [48].

Considering the intensity and frequency of the interventions, this again ranged greatly. Intensity varied from very light to near maximal and frequency varied from a single Qigong session (6 minutes long) [69] to 18 months of 15 minutes daily arm exercises [96]. Interventions were most frequently (n = 20; 26.6%) reported as 12 weeks/ 3 months in length (S6 Table).

To further understand exercise prescription, the 79 interventions were mapped against CERT [37] (S2 Table). This highlighted that interventions were most likely to be supervised (n = 34; 43%) [41–43,45,50–55,57,59–61,63,71–73,80,82,88,93,95,97,98,101–103,107–109,112,113,116], in a group (n = 23; 29.1%) [41–43,50–52,54,55,60,63,75,80,85,87,97,98,101,107,108,112–114,116] by a Physiotherapist (n = 25; 31.6%) [40,46,50–52,55,56,63,69,70,72–75,80,81,83,96,98,101,102,107,112–114] and carried out in a research facility such as a hospital, clinic, rehabilitation unit, university or research centre (n = 27; 34.2%) [40,46,48–50,52,53,55,56,58,59,63,65,69,71–73,76,83,84,88,90,91,100,102,107,114]. Compression to the arm, either as a compression armsleeve or compression bandage, at participants choice or mandatory, was the most frequently included non-exercise component (n = 31; 39.2%) [44,45,47,49,55,57–60,62,64,68,72,73,75,76,78,80,81,87,89,93,96,98,104–108,110,118]. 53.2% (n = 42) of the studies provided a detailed description of how adherence to the intervention was measured and reported and 34.2% (n = 27) gave a detailed description of any motivational strategies incorporated (S2 Table).

The rationale upon which the PA interventions were designed was also charted. This demonstrated that the majority were developed upon the scientific rationale of previous studies (n = 50; 63.3%) and/or the physiological rationale that PA encourages muscle-pump action and lymph drainage (n = 34; 43%) (S3 Table). 8 studies (10.1%) included the ACSM exercise guidelines for cancer survivors [121] in their design of [51,60,62,63,67,88,101,103] and only five studies reported including a traditional behavioural change theory: the social cognitive theory (n = 4; 5.1%) [50,104–106] and transtheoretical model (n = 1; 1.3%) [74].

### Outcome measures

S6 Table provides a summary of the lymphoedema outcomes and other main outcomes measured. There were seven objective measures of lymphoedema reported. These included: bioimpedance spectrometry (BIS), water displacement, perometry, dual-energy x-ray absorptiometry (DXA), tissue tonometry, ultrasound (US) and the most common, arm circumference (n = 43; 54.4%). 53.2% (n = 42) of the studies also included a self-report measure of BCRL, however, only 8.9% (n = 7) included clinical examination of the affected limb as a reported outcome [46,53,96,104–106,117].

Other outcomes mostly commonly measured included: QOL 48.1% (n = 38); strength 40.5% (n = 32); ROM 22.8% (n = 18); function/disability 26.6% (n = 21); pain 20.3% (n = 16); fatigue 6.3% (n = 5); anxiety/depression/mood 6.3% (n = 5).

Intervention acceptability was only reported in 17.7% (n = 14) of the studies. This was through comments received (n = 4) [70,76,93,113], questionnaire evaluation (n = 5) [40,58,62,63,111] or qualitative enquiry (n = 5) [50,56,57,75,89]. Where reported, this feedback was both positive, e.g., “participants reported more confidence in using the affected upper limb …. they were less often reminded of having lymphedema” [70] and negative, e.g., “….feeling of getting cold when only exercising at slower speeds ….preferred more active classes” [63].

### Participants’ volumes and patterns of PA

Less than a quarter of the studies recorded PA levels of the participants at baseline (21.5%; n = 17) via various different self-report measures such as the International Physical Activity Questionnaire (IPAQ) (n = 3; 3.8%) [40,104,105] (S6 Table). However, where published (6.3%; n = 5) most recorded participants as active [46,57,62,65,78]. Throughout the intervention, two studies measured daily step activity of the main intervention group via a smart bracelet [67,101] and one via a pedometer [47].

At the end of the intervention 8.9% (n = 7) of the studies reported if PA levels had differed from baseline. No significant change in PA level was reported in three studies [95,104,105] whereas in contrast, three studies reported an increase in PA levels [51,76,101]. In another study despite an initial increase in PA level at 3 and 6 months, this increase was not sustained at 2years [102].

## Discussion and Areas for Further Research

The traditional approach to PA research to prevent and manage BCRL has focused on the safety and effectiveness of PA on lymphoedema outcomes. Thus, clinical practice guidelines now recommend that PA is safe for this population [13]. However, in order to further understand how research in this area could be translated into real-life scenarios, this scoping review explored the core components of 79 PA intervention studies for those at risk of, or living with, BCRL from across the globe.

The participants most frequently included in the interventions were 50–60 years old females (58.2%), diagnosed with stage II breast cancer (21.5%), had received the breast cancer treatments axillary node surgery (81%), radiotherapy (69.6%) and chemotherapy (68.4%), and were overweight or obese (65.8%). While these demographics represent those most at risk of breast cancer and BCRL [122,123], reflecting those easiest to recruit to such studies, it does highlight gaps within the literature with regarding understudied populations. For example, around 390 men are diagnosed with breast cancer each year in the UK and this population was not adequately represented [122].

BCRL risk is also associated with the extent of lymph node removal, site of radiotherapy [2] and the time since breast cancer diagnosis and treatment [124]. Most studies recruited participants less than 6 months post diagnosis or surgery (26.6%) or beyond 60 months (5 years) post active treatment, surgery or diagnosis (26.6%). This may be attributed to studies investigating lymphoedema prevention recruiting participants early in the cancer timeline, i.e., before BCRL develops and studies investigating lymphoedema management recruiting participants later in the cancer timeline, i.e., when there is a confirmed diagnosis of BCRL. However BCRL risk is reported to peak between 12 and 30 months postoperatively [124]. Therefore, future PA and BCRL research would benefit from longitudinal studies, recruiting participants from time of diagnosis and following them up for a number of years post treatment with consideration given to lesser researched risk factors, e.g., the extent of lymph node removal and understudied populations.

With the historical assumption that over-exertion of the affected limb could result in swelling [10], it is not surprising that in the attempt to prove that PA is safe for those at risk of, or living with BCRL, the majority of studies included in this scoping review focused on supervised (43%), upper body RE (25.3%), carried out in a research type facility (34.2%) as the main mode of enquiry. Similarly in a study of 48 BCS with BCRL the majority (79.1%) reported that they prefer exercise to be supervised however, in contrast the majority preferred this to be in a clinic/sports centre (50%) and the type of PA to be walking or jogging (66.6%) [12]. With 50% of this population self-reporting as inactive [12], future research would benefit from not only considering the PA preferences of this population but also investigating how to reduce sedentary time to promote positive survivorship.

The inclusion of the CERT into this scoping review identified that 34.2% of the studies included motivational strategies to encourage PA participation which is unsurprising given the limited use of behaviour change theory in the design of just five studies (6.3%). With most interventions instead based primarily upon scientific (63.3%) and/or physiological rationale (43%) it is important to recognise that PA behaviour has been negatively associated with a BCRL diagnosis. For example, in a 5 year longitudinal study, PA levels (self-reported as regular, occasional or none) of BCS were found to be significantly (p = 0.004) lower in those with a BCRL diagnosis when compared to those without a BCRL diagnosis [125]. These findings, alongside the infrequent reporting of intervention acceptability (17.7%) and the inconsistent reporting of PA levels at baseline or throughout the studies, highlights a limited understanding of PA behaviours amongst BCS at risk of, or living with, BCRL. Future research that incorporates behavioural change theory would enable better understanding of long-term PA behaviour in this population.

The outcomes measured in the included studies ranged from seven different objective lymphoedema measures to several outcomes associated with common cancer treatment-related side effects, e.g., strength. This range reflects the multifaceted outcomes and benefits associated with PA after breast cancer treatment. Arm circumference was the most popular objective lymphoedema outcome measure (54.4%) most likely due to its wide availability and low cost [126]. However, how best to measure, diagnose, and monitor BCRL remains the subject of international debate [127] with the heterogeneity in lymphoedema outcome measures cited as a limitation in a previous PA and BCRL systematic review [16]. One suggestion has been that to avoid BCRL misdiagnosis in up to 50% of BCS, BCRL diagnosis should include not only an objective measurement but also self-reported symptoms and clinical examination [128]. Whilst self-report measures were used in 53.2% of the included studies, clinical examination was reported much less frequently, in only 8.9% of the studies. Future research would benefit from addressing the diversity of outcomes and gaining international agreement on standardised lymphoedema outcomes.

### Strengths and Limitations

Incorporating both structured and unstructured PA interventions into this scoping review enabled a comprehensive overview of both PA and exercise research in this area. With the majority of interventions being structured, thus resembling exercise, the CERT template was consulted to provide clarity in the data charting. Implementing the CERT in a scoping review is a novel method to aid with data charting of interventions in this area. Thus, research gaps around motivational strategies were identified. Another novel approach used in this study was utilising the ACSM classification of exercise intensity to categorise exercise intensity and this highlighted the sheer breadth of intensities and terminology used throughout these interventions.

Limitations of this scoping review include limiting the search strategy to the English language. The authors recognise the worldwide attention this topic receives however, this limitation was unavoidable due to language and time constraints of the authors. One study was excluded due to the data reported not being sufficient enough to chart. Despite best efforts, the author did not respond to contact. Although 79 other studies were charted, this is recognised as a limitation. Another limitation of this scoping review is the consideration of diverse populations. Whilst the gender of participants was explored, highlighting the omission of males from most studies, other demographics such as socioeconomic factors were not explored due to the volume of data to be chartered.

Also lymphoedema was not the main focus of all studies, but a secondary outcome of a larger study. This was not always clearly reported and difficult to ascertain at times. To try and fully understand the interventions, the authors searched for accompanying articles where available (e.g., study protocols or studies reporting other outcomes from the same study).

## Conclusion

PA interventions for those at risk of, or living with, BCRL focus on short-term goals such as preventing lymphoedema or reducing lymphoedema arm volume. Research in this area is therefore commonly designed upon scientific or physiological rationale, includes populations easiest to recruit, is limited in duration and focuses on objective lymphoedema outcomes. Future research recommendations to encourage long-term PA behaviour are summarised below.

### Recommendations

In summary this scoping review highlighted five gaps and recommendations for future research. These should be addressed to help bridge the gap between research informed clinical guidelines and real-world situations. These include:

1. Involve stakeholders early on in the research process. In particular recognising the PA preferences and behaviours of those at risk of, and living with, BCRL.
2. Recognise the impact risk factors for lymphoedema may have on BCRL severity and PA behaviours.
3. Target research early in the cancer treatment and survivorship timeline, when participants are most susceptible to BCRL development or progression.
4. Identify effective behavioural change theories to enhance long-term PA participation in this population.
5. Collaborate with stakeholders to investigate how best to diagnose and measure BCRL beyond objective measures alone.

## Supporting information

S1 Table PRISMA Checklist. Preferred reporting items for systematic reviews and meta-analyses extension for scoping reviews (PRISMA-ScR) checklist.
(PDF)

S2 Table CERT checklist. Consensus on Exercise Reporting Template.
(XLSX)

S3 Table Study characteristics. Characteristics of 79 studies included.
(PDF)

S4 Table Grey literature. Relevant home pages for key societies and professional organisations.
(PDF)

S5 Table CINAHL search strategy. The search strategy from the database CINAHL.
(PDF)

S6 Table Study Summary. [40–71,73–118]. Abbreviations: Resistance exercise (RE); Aerobic exercise (AE); Quality of life (QOL); Range of motion (ROM); Breast cancer specific quality of life questionnaire module of the European Organisation for Research and Treatment for Cancer (BR23); Bioimpedance Spectrometry (BIS); Metabolic Equivalent of Task (MET); International Physical Activity Questionnaire (IPAQ); Control group (CG); Intervention group (IG); Physical activity (PA); Community Healthy Activities Model Program for Seniors (CHAMPS). Visual analogue scale (VAS). Numerical rating scale (NRS). Dual-energy X-ray Absorptiometry (DXA). Lymphoedema and breast cancer questionnaire (LBCQ). Functional assessment of cancer therapy- breast cancer + arm subscale (FACT-B + 4). Body mass index (BMI). Breast cancer-related lymphoedema (BCRL). Ultrasound (US). Note: For the randomised controlled trials (RCT) group A is the intervention group and Group B is the control group except when there is more than one intervention group. In this case if three groups are reported Group A, Group B are the intervention group and Group C is the control group. If four groups are reported then Group A, Group B, Group C are the intervention group and Group D is the control group.
(DOCX)

S1 Fig Cancer staging. The most frequently reported cancer stage of included participants per study.
(TIF)

S2 Fig Treatment type. Graph to show the percentage of studies that reported participants received each type of breast cancer treatment.
(TIF)
