# Supplementary material for: Core components of physical activity interventions in the prevention and management of breast cancer-related lymphoedema (BCRL): A scoping review
Source: PLoS One. 2026 Sep 2;21(9):e0355189. doi: 10.1371/journal.pone.0355189 (PMC13537585; doi:10.1371/journal.pone.0355189)
Supplement: Supplementary file 1 [file pone.0355189.s001.pdf]

S1 Table- PRISMA-ScR checklist

Adapted from: Tricco AC, Lillie E, Zarin W, O'Brien KK, Colquhoun H, Levac D, et al. PRISMA Extension for Scoping Reviews (PRISMA-ScR): Checklist and Explanation. Ann Intern Med. 2018;169(7):467–73. https://doi.org/10.7326/M18-0850

Section	PRISMA-ScR Guideline	Checklist
Title	Identify the paper as a scoping review	Page 1
Abstract		
Structured summary	Provide a structured summary that includes (as applicable): background, objectives, eligibility criteria, sources of evidence, charting methods, results, and conclusions that relate to the review questions and objectives.	Page 2-3
Introduction		
Rationale	Describe the rationale for the review in the context of what is already known. Explain why the review questions/objectives lend themselves to a scoping review approach.	Pages 3-5
Objectives	Provide an explicit statement of the questions and objectives being addressed with reference to their key elements (e.g. population or participants, concepts, and context) or other relevant key elements used to conceptualize the review questions and/or objectives.	Pages 5-6
Methods		
Protocol and registration	Indicate whether a review protocol exist: state if and where it can be accessed (e.g. a Web address); and if available, provide registration information, including the registration number.	Page 6
Eligibility criteria	Specify characteristic tics of the sources of evidence used as eligibility criteria (e.g., years considered, language, and publication status), and provide a rationale.	Pages 6-7
Information sources	Describe all information sources in the search (e.g., databases with dates of coverage and contact with authors to identify additional sources), as well as the date the most recent search was executed.	Pages 7-8
Search	Present the full electronic search strategy for at least 1 database, including any limits used, such that it could be repeated.	Table 1. S5.
Selection of sources of evidence	State the process for selecting sources of evidence (i.e., screening and eligibility) included in the scoping review.	Page 9
Data charting process	Describe the methods of charting data from the included sources of evidence (e.g., calibrated forms or forms that have been tested by the team before their use, and whether data charting was done independently or in duplicate) and any processes for obtaining and confirming data from the investigators.	Page 9
Data items	List and define all variables for which data were	Page 9-10

	sought and any assumptions and simplifications made.	
Critical appraisal of individual sources of evidence	If done, provide a rationale for conducting a critical appraisal of included sources of evidence; describe the methods used and how this information was used in any data synthesis of appropriate.	N/A
Synthesis of results	Describe the methods of handling and summarizing the data that were charted.	Page 10
Results		
Selection of sources of evidence	Give numbers of sources of evidence screened, assessed for eligibility, and included in the review, with reasons for exclusions at each stage, ideally using a flow diagram.	Page 11. Figure 1
Characteristics of sources of evidence	For each source of evidence, present characteristics for which data were chartered and provide the citations.	Page 12. Table 2.
Critical appraisal within sources of evidence	If done, present data on critical appraisal of included sources of evidence (see item 2).	N/A
Results of individual sources of evidence	For each included source of evidence, present the relevant data that were charted that relate to the review questions and objectives.	Pages 12-33. Table 2. Table S2. S3. Figure. S1. S2.
Synthesis of results	Summarize and/or present the charting results as they relate to the review questions and objectives.	Pages 34-38
Discussion		
Summary of evidence	Summarize the main results (including an overview of concepts, themes, and types of evidence available), link to the review questions and objectives, and consider the relevance to key groups.	Pages 381-4
Limitations	Discuss the limitations of the scoping review process.	Pages 41-42
Conclusions	Provide a general interpretation of the results with respect to the review questions and objectives, as well as potential implications and/or next steps.	Pages 42-43
Funding		
Funding	Describe sources of funding for the included sources of evidence, as well as sources of funding for the scoping review. Describe the role of the funders of the scoping review.	Financial disclosure statement
