# Supplementary material for: Core components of physical activity interventions in the prevention and management of breast cancer-related lymphoedema (BCRL): A scoping review
Source: PLoS One. 2026 Sep 2;21(9):e0355189. doi: 10.1371/journal.pone.0355189 (PMC13537585; doi:10.1371/journal.pone.0355189)
Supplement: Supplementary file 2 [file pone.0355189.s003.pdf]

S3 Table: Study Characteristics (n=79)

| | Author (Year) | Country | Manage/ Prevent /Both | Pilot/ Feasibility | Funding | Scientific background | Theoretical background |
|---|---|---|---|---|---|---|---|
| Randomised controlled trials (n=44) | | | | | | | |
| 1 | McKenzie et al. (2003) | Canada | Manage | No | | Previous study | |
| 2 | Kilbreath et al. (2006) | Australia | Prevent | No | | Previous study | |
| 3 | Ahmed et al. (2006) | USA | Both | No | Charity; Government; University | Physiological basis | |
| 4 | Todd et al. (2008) | UK | Prevent | No | | Previous study. Physiological basis | |
| 5 | Hayes et al. (2009) | Australia | Manage | No | Charity | Previous study. Australian Institute for Health and Welfare guidelines (2004) | |
| 6 | Schmitz et al. (2009) | USA | Manage | No | Charity; Government; Private; University | Previous study | Social Cognitive Theory |
| 7 | Tidhar et al. (2010) | Israel | Manage | No | | Previous study. Physiological basis | |
| 8 | Schmitz et al. (2010) | USA | Prevent | No | Charity; Government; Private; University | Previous study | Social Cognitive Theory |
| 9 | Malicka et al. (2011) | Poland | Both | No | | Physiological basis | |
| 10 | Kilbreath et al. (2012) | Australia | Prevent | No | Charity; Government; Private | Physiological basis | |
| 11 | Anderson et al. (2012) | USA | Prevent | No | Government | ACS lymphoedema workshop guidelines (Rockson, 1998) | |
| 12 | Jeffs and Wiseman (2013) | UK | Manage | Yes | Charity | Previous study. Leduc MLD method | |



Abbreviations: ACS, American Cancer Society; MLD, Manual Lymphatic Drainage; USA, United States of America; UK, United Kingdom; ACSM, American College of Sports Medicine; PA, Physical Activity; BASES, British Association of Sports and Exercise Sciences.

| | | | | | | | |
|---|---|---|---|---|---|---|---|
| 13 | Johansson et al. (2013) | USA | Manage | Yes | Charity | Previous study. Physiological basis | |
| 14 | Cormie et al. (2013b) | Australia | Manage | No | Government; University | Physiological basis. ACS (Rock, 2012) and ACSM (2010) guidelines | |
| 15 | Letellier et al. (2014) | Canada | Manage | Yes | Charity | Physiological basis. Casley Smith method (1997) | |
| 16 | Loudon et al. (2014) | Australia | Manage | Yes | University | Previous study. Physiological basis. Holistic benefits of yoga | |
| 17 | Do et al. (2015) | South Korea | Manage | No | | Previous study. Physiological basis | |
| 18 | DiBlasio et al. (2016) | Italy | Manage | No | | Physiological basis | |
| 19 | Zhang et al. (2016) | China | Prevent | No | Government | National Lymphoedema Network Position paper | |
| 20 | Şener et al. (2017) | Turkey | Manage | No | None | Previous study | |
| 21 | Schmidt et al. (2017) | Germany | Both | No | Private | Previous study. ACSM (2010) guideline | |
| 22 | Donmez and Kapucu (2017) | Turkey | Prevent | No | University | Previous study | |
| 23 | Ammitzbøll et al. (2019) | Denmark | Prevent | No | Charity; Private | Previous study | |
| 24 | Bloomquist et al. (2019) | Denmark | Both | No | Charity | Previous study. Physiological basis. Danish Health and Medicines Authority: PA guidelines (2013) | Social Cognitive Theory |
| 25 | Odynets et al. (2019) | Ukraine | Manage | No | None | Previous study | |
| 26 | Schmitz et al. (2019) | USA | Manage | No | Charity; Government; Private; University | Previous study | Social Cognitive Theory |



Abbreviations: ACS, American Cancer Society; MLD, Manual Lymphatic Drainage; USA, United States of America; UK, United Kingdom; ACSM, American College of Sports Medicine; PA, Physical Activity; BASES, British Association of Sports and Exercise Sciences.

| | | | | | | | |
|---|---|---|---|---|---|---|---|
| 27 | Pasyar et al. (2019) | Iran | Manage | Yes | University | Physiological basis. Benefits of yoga | |
| 28 | Kilbreath et al. (2020) | Australia | Manage | No | Government | Previous study | |
| 29 | Omar et al. (2020) | Egypt | Manage | No | | Previous study | |
| 30 | Naughton et al. (2021) | USA | Prevent | No | Government | Previous study. ACS for lymphedema care/prevention) | |
| 31 | Bruce et al. (2021) | UK | Prevent | No | Government; University | ACS guidelines (Rock, 2012) | |
| 32 | Bloomquist et al. (2021) | Denmark | Both | No | Charity | Previous study | |
| 33 | Ali et al. (2021) | Egypt | Manage | No | | Previous study. The National Lymphoedema Network position statement | |
| 34 | Lin et al. (2022) | China | Prevent | No | Government | ACSM (2019) China Anti-Cancer Association guidelines | |
| 35 | Naczk et al. (2022) | Poland | Prevent | No | None | Physiological basis | |
| 36 | Balaji and Senthil (2024) | India | Manage | Yes | | Previous study. Cancer rehabilitation principles | |
| 37 | Venugopalan et al. (2024) | India | Prevent | Yes | | Previous study | |
| 38 | Harvie et al. (2024) | UK | Manage | Yes | Charity | Physiological basis. BASES Expert Statement on exercise and cancer survivorship | Transtheoretical model |
| 39 | Esteban-Simon et al. (2024) | Spain | Both | No | Government; University | Previous study. ACSM (2019) and walking guidance. ACS (for people at risk of lymphoedema (2021). National Cancer Institution recommendations | |



Abbreviations: ACS, American Cancer Society; MLD, Manual Lymphatic Drainage; USA, United States of America; UK, United Kingdom; ACSM, American College of Sports Medicine; PA, Physical Activity; BASES, British Association of Sports and Exercise Sciences.

| | | | | | | | |
|---|---|---|---|---|---|---|---|
| 40 | Nakamoto et al. (2024) | Japan | Prevent | No | Charity; Private | Previous study. Japanese Breast Cancer Society Clinical Practice Guidelines for cancer treatment | |
| 41 | Giron et al. (2025) | Brazil | Both | No | | Previous study | |
| 42 | Maraş Baydogan et al. (2025) | Turkey | Prevent | No | None | Previous study | |
| 43 | Ramirez-Parada et al. (2025) | Chile | Prevent | No | | Previous study. ACSM (2019) guidelines. | Chronic Care Model |
| 44 | Zhang et al. (2025) | China | Prevent | No | Charity; Government | Previous study. ACSM Resistance training principle guidelines. | |
| Single Group (n=15) | | | | | | | |
| 45 | Lane et al. (2005) | Canada | Prevent | No | Charity | Previous study | |
| 46 | Johansson and Piller (2007) | Australia | Manage | Yes | | Previous study | |
| 47 | Jönsson and Johansson (2009) | Sweden | Manage | No | Charity | Physiological basis. | |
| 48 | Gautam et al. (2011) | India | Manage | No | None | Previous study | |
| 49 | Stan et al. (2012) | USA | Prevent | Yes | Charity | Physiological basis | |
| 50 | Jönsson and Johansson (2014) | Sweden | Manage | Yes | | Previous study. Physiological basis. ACSM (1998) recommendations. | |
| 51 | Fisher et al. (2014) | USA | Manage | Yes | University | Previous study. Physiological basis. | |
| 52 | Yen-TingLai et al. (2017) | Taiwan | Both | Yes | Government | Previous study. Physiological basis. | |



Abbreviations: ACS, American Cancer Society; MLD, Manual Lymphatic Drainage; USA, United States of America; UK, United Kingdom; ACSM, American College of Sports Medicine; PA, Physical Activity; BASES, British Association of Sports and Exercise Sciences.

| | | | | | | | |
|---|---|---|---|---|---|---|---|
| 53 | Godoy et al. (2017) | Brazil | Prevent | No | | Physiological basis | |
| 54 | Simonavice et al. (2017) | USA | Both | No | University | ACSM (2014 exercise prescription) guidelines | |
| 55 | Ammitzboll et al. (2017) | Denmark | Prevent | Yes | Charity | Physiological basis | |
| 56 | Mazor et al. (2018) | USA | Prevent | No | University | Physiological basis. Mental benefits of yoga. | |
| 57 | Salacinski et al. (2021) | USA | Prevent | No | Charity | Physiological basis. ACS (unknown), ACSM (Nelson, 2007), Susan G. Komen BC Foundation, National Lymphoedema Network. | |
| 58 | Carretti et al. (2022) | Italy | Manage | Yes | Government; University | Physiological basis | |
| 59 | Pardo-Hernandez et al. (2024) | Mexico | Both | No | None | Previous study. Physiological basis. ACSM exercise prescription guidance. | |
| Randomised cross-over trial (n=8) | | | | | | | |
| 60 | Johannsson et al. (2005) | Sweden | Manage | No | Charity | Previous study | |
| 61 | Bracha and Katz-Leurer (2012) | Israel | Manage | No | Charity | Physiological basis | |
| 62 | Cormie et al. (2013a) | Australia | Manage | No | Government; University | Physiological basis | |
| 63 | Singh et al. (2015) | Australia | Manage | No | Government | Previous studies. National Lymphoedema Network Guidelines (2013) | |
| 64 | Cormie et al. (2016) | Australia | Manage | No | Government | Physiological basis | |



Abbreviations: ACS, American Cancer Society; MLD, Manual Lymphatic Drainage; USA, United States of America; UK, United Kingdom; ACSM, American College of Sports Medicine; PA, Physical Activity; BASES, British Association of Sports and Exercise Sciences.

| | | | | | | | |
|---|---|---|---|---|---|---|---|
| 65 | Bloomquist et al. (2018) | Denmark | Prevent | No | University | Previous study | |
| 66 | Deacon et al. (2019) | Australia | Both | No | None | Physiological effects. Lymphoedema Framework - Best Practice for the management of lymphoedema International Consensus Document (2006)<br>ACSM guidelines (2010). | |
| 67 | Guloren et al. (2023) | Turkey | Manage | No | None | Previous study. Physiological basis | |
| Non-randomised controlled trial (n=8) | | | | | | | |
| 68 | Moseley et al. (2005) | Australia | Manage | No | | Previous study | |
| 69 | Fong et al. (2014) | Hong Kong | Manage | Yes | None | Previous study. Physiological basis. | Qigong psycho neuroimmunology theory |
| 70 | Buchan et al. (2016) | Australia | Manage | No | None | Previous study | |
| 71 | Singh et al. (2016) | Australia | Manage | No | | Previous study. National Lymphoedema Network guidelines (2013). | |
| 72 | Lacorossi et al. (2019) | Italy | Both | No | | Previous study | Functional-descriptive model of posttraumatic growth |
| 73 | Atef et al. (2020) | Egypt | Manage | No | None | Physiological basis | |
| 74 | Boeer et al. (2023) | Germany | Both | No | Charity | Previous study | |
| 75 | Damato et al. (2023) | Russia | Manage | No | | Previous study. ACSM guidelines (2010) | |
| Cohort study (n=2) | | | | | | | |
| 76 | Douglass et al. (2012) | Australia | Manage | No | Charity | Casley-Smith lymphoedema exercise guidelines (1999) | |



Abbreviations: ACS, American Cancer Society; MLD, Manual Lymphatic Drainage; USA, United States of America; UK, United Kingdom; ACSM, American College of Sports Medicine; PA, Physical Activity; BASES, British Association of Sports and Exercise Sciences.

| | | | | | | | |
|---|---|---|---|---|---|---|---|
| 77 | Bloomquist et al. (2014) | Denmark | Prevent | No | | ACSM guideline (2010) | |
| Other (n=2) | | | | | | | |
| 78 | Tidhar et al. (2004) | Israel | Manage | No | | Physiological basis | |
| 79 | Sagen et al. (2009) | Norway | Prevent | No | Charity; Government | Previous study | |



Abbreviations: ACS, American Cancer Society; MLD, Manual Lymphatic Drainage; USA, United States of America; UK, United Kingdom; ACSM, American College of Sports Medicine; PA, Physical Activity; BASES, British Association of Sports and Exercise Sciences.
