# Supplementary material for: Core components of physical activity interventions in the prevention and management of breast cancer-related lymphoedema (BCRL): A scoping review
Source: PLoS One. 2026 Sep 2;21(9):e0355189. doi: 10.1371/journal.pone.0355189 (PMC13537585; doi:10.1371/journal.pone.0355189)
Supplement: Supplementary file 3 [file pone.0355189.s004.pdf]

S4 Table – Grey literature search of identified professional body and relevant charity websites

The following list was developed on 29-11-2024 and the websites searched for relevant information and studies relating to physical activity and breast cancer related lymphoedema.

Professional body or charity	Website
American Cancer Society	www.cancer.org
Lymphology Association of North America	www.clt-lana.org
National Cancer Institute	www.cancer.gov/cancertopics/pdq/supportivecare/lymphedema
National Lymphedema Network	www.lymphnet.org
British Lymphedema Society	thebls.com
Canadian Lymphedema Framework	canadalymph.ca
European Society of Lymphology	eurolymphology.org
Földi Klinik	foeldiklinik.de
International Lymphoedema Framework	lympho.org
Lymphedema Association of Ontario	lymphontario.ca
Breast Cancer Foundation NZ	www.breastcancerfoundation.org.nz
Lymphatic Education & Research Network	http://lymphaticnetwork.org/
Lymphedema Seminars	http://lymphseminars.com/
Australasian Lymphology Association	http://www.lymphoedema.org.au/
Cancer Research	www.cancerresearchuk.org
Breast Cancer Now	Breastcancernow.org
Macmillan	www.macmillan.org.uk
Restore scar therapy	Restore Therapy: The leading scar therapy educator in UK
