# Supplementary material for: Core components of physical activity interventions in the prevention and management of breast cancer-related lymphoedema (BCRL): A scoping review
Source: PLoS One. 2026 Sep 2;21(9):e0355189. doi: 10.1371/journal.pone.0355189 (PMC13537585; doi:10.1371/journal.pone.0355189)
Supplement: Supplementary file 4 [file pone.0355189.s005.pdf]

S5 Table – CINAHL search strategy

S1	(MH "Exercise+") OR (MH "Athletic Training") OR (MH "Physical Therapy+") OR (MH "Activities of Daily Living+") OR (MH "Rehabilitation+")
S2	(MH "Leisure Activities+")
S3	(MH "Sports+")
S4	S1 OR S2 OR S3
S5	"Physical Activ*" or PA or Activ* or Exercis* or Housework or ADL* or Recreation or Danc* or Garden* or Aerobic* or Fitness or "Physical Endurance" or "Activ* Energy Expenditure*" or "Caloric Expenditure*" or "Human Physical Conditioning" or "Circuit Train*" or "Circuit-Based Train*" or "Circuit Based Train*" or Plyometric or Stretch* or Drill* or "Strength Train*" or "Resist* Train*" or "Weight* Lift*" or Weight-Lift* or Weightlift* or Bicycl* or Bike or Biking or Cycl* or Walk* or Ambulat* or Run* or Jog* or Stand* or "Aqua* Therapy" or Swim* or Row* or Yoga or "Tai Chi" or Pilates or Inactiv* or "Sedent* behavio?r*" or "Sedent* lifestyle*" or "Stationary behavio?r*" or "Screen time" or "Health behavio?r" or "Health Promotion" or "Body Weight Change*" or "Weight Reduction Program*" or "Weight Gain Prevention" or "Body weight maintenance" or Sport*
S6	(MH "Lymphedema") AND (MH "Breast Neoplasms+")
S7	"Breast cancer* lymph?edema" or "Breast neoplasm* lymph?edema" or "breast carcinoma* lymph?edema" or "breast tum?ur* lymph?dema" or "breast malignanc* lymph?edema" or "Postmastectomy lymph?edema" or "post-mastectomy lymph?edema" or "post mastectomy lymph?edema" or "breast cancer related lymph?edema" or "breast cancer-related lymph?edema" or "breast cancer-treatment lymph?edema" or "breast cancer treatment lymph?edema" or BCRL
S8	S4 OR S5
S9	S6 OR S7
S10	S8 AND S9
Additions	Limited to English Language<br>Date 01/01/2000 – 12/31/2024<br>Proximity<br>Apply equivalent subjects
Results	n=611
