# Supplementary material for: Core components of physical activity interventions in the prevention and management of breast cancer-related lymphoedema (BCRL): A scoping review
Source: PLoS One. 2026 Sep 2;21(9):e0355189. doi: 10.1371/journal.pone.0355189 (PMC13537585; doi:10.1371/journal.pone.0355189)
Supplement: Supplementary file 5 [file pone.0355189.s006.docx]

| **Author**  **Year** | **Country** | **Sample size** | | | | | **Physical Activity Intervention** | | **Timeline** | **Outcomes** | | **Findings** | | |
| --- | --- | --- | --- | --- | --- | --- | --- | --- | --- | --- | --- | --- | --- | --- |
|  |  | **Baseline age in years: Mean ± SD OR Median (IQR)** | | | | | **Type/ Time/ Frequency** | **Intensity** |  | **Lymphoedema** | **Other outcomes of interest** | **Lymphoedema** | **Other** | |
|  |  | **Baseline BMI (kg/m²): Mean ± SD OR Median (IQR)** | | | | |  |  |  |  |  |  |  |  |
|  |  | **Total** | **Group A** | **Group B** | **Group C** | **Group D** |  |  |  |  |  |  |  |  |
| Randomised controlled trials (n=44) | | | | | | | | | | | | | |  |
| McKenzie et al.  (2003) (93) | Canada | 14 | 7 | 7 |  |  | **RE of upper body.** Compression sleeve. 2-3reps x 10.  Warm up/cool down/stretching. Upper body AE added after 2 weeks.  3 x per week x 8 weeks. | Light and progressed | > 6months post active treatment | Circumference.  Water displacement. | QOL.  Feedback comments. | ↔ No significant change in arm volume between groups over time. | ↔ No significant difference in QOL between groups across time. | |
|  |  |  | 56.4±10.4 | 56.9 ±8.2 |  |  |  |  |  |  |  |  |  |  |
|  |  |  | 29.1 ±6.6 | 25.6 ±3.3 |  |  |  |  |  |  |  |  | *↑ Anecdotal subjective improvement in the health of the affected arm in ways not measured.* | |
| Kilbreath et al.  (2006) (83) | Australia | 22 | 14 | 8 |  |  | **RE and ROM exercises of upper body plus usual care.**  RE: 8-12reps x 2. Stretch: held 5-15mins.  Daily x 8 weeks. | Moderate | < 6 weeks post-surgery | Circumference.  Self-report (BR23) | QOL.  Strength.  ROM. | ↑Greater proportion of women in the control group had an interlimb difference ≥2cm when compared to those in the exercise group.  ↑Women in the exercise group had fewer arm and breast symptoms compared to the control. | ↔ Whilst interlimb differences in strength and ROM for the exercise group had less of a difference, these were not significant. | |
|  |  | 52 ±12 | 52.7 ±14 | 51.5 ±10.2 |  |  |  |  |  |  |  |  |  |  |
|  |  |  |  |  |  |  |  |  |  |  |  |  |  |  |
| Ahmed et al.  (2006) (44) | USA | 45 | 23 | 22 |  |  | **RE and ROM stretches of upper and lower body** x up to 3sets x 60min.  Plus normal lymphoedema self-care.  2 x per week x 6months. | Light and progressed | > 12months post active treatment (Mean) | Circumference. Self-report (Norman questionnaire). | Strength.  Baecke Questionnaire. | ↔ Neither the incidence of lymphedema nor the onset of lymphedema symptoms varied in the intervention-group versus control-group participants. | ↔ Covariates of Baecke Questionnaire examined but not included in final model. | |
|  |  |  | 52.3 ±7.7 | 51.7 ±7.5 |  |  |  |  |  |  |  |  |  |  |
|  |  |  | 27.1 ±4.9 | 26.5 ±4.9 |  |  |  |  |  |  |  |  |  |  |
| Todd et al.  (2008) (114) | UK | 116 | 58 | 58 |  |  | **Arm/shoulder exercises x 10min.**  4 x per day until full shoulder ROM restored.  Then 1 x per x 1 year. |  | Recruited at diagnosis, undergoing surgery. | Water displacement. Self-report (telephone questionnaire). | QOL.  Strength.  ROM.  Function/  disability. | ↑ Delayed shoulder mobilisation resulted in smaller limb volume differences and reduced incidence of lymphoedema when compared with early shoulder mobilisation. | ↔ No statistically significant differences in secondary outcomes. ↓ Self-reported swelling can have a detrimental effect on QOL. | |
|  |  | 57 ±13.1 | 56.5 ±12.4 | 57.2 ±14 |  |  |  |  |  |  |  |  |  |  |
|  |  |  | 27.5 ±5.6 | 28.4 ±5.7 |  |  |  |  |  |  |  |  |  |  |
| Hayes et al.  (2009) (75) | Australia | 32 | 16 | 16 | 106 Nonparticipants |  | **AE (weeks 1-12) of all types and RE (weeks 2-12) of upper and lower body.** Progressing over 12 weeks  20min - 45+min x 3-4 per week. | Light and progressed | >60months post active treatment (Mean) | Perometry.  BIS.  Self-report (questionnaire). | Qualitative feedback. | ↔ No statistically significant changes in lymphoedema for the IG over the study period. | *↓ One overarching concern 'lymphedema impacts all facets of an individual's life'.* | |
|  |  | 59 ±9 | 60 ±11 | 59 ±7 | 60 ±10 |  |  |  |  |  |  |  |  |  |
|  |  |  |  |  |  |  |  |  |  |  |  |  |  |  |
| Schmitz et al. (2009) (105) | USA | 141 | 71 | 70 |  |  | **RE of upper and lower body** x 2-3 sets x 10 x 90mins.  AE warm-up.  2 x per week x 12months. | Very light and progressed | >60months post diagnosis (Mean) | Water displacement. BIS.  Self-report (Norman questionnaire). Clinical examination. | QOL.  Strength.  ROM.  Function.  Pain. Anxiety/Depression.  Fatigue.  IPAQ. | ↔ No significant effect on the interlimb volume ≥5%, between groups.  ↑ The number and severity of symptoms reported decreased more in the weight-lifting group than in the control group. | ↑ Significant improvements in upper and lower body strength. | |
|  |  |  | 56 ±9 | 58 ±10 |  |  |  |  |  |  |  |  |  |  |
|  |  |  | 31.0 ±6.2 | 29.9 ±6.6 |  |  |  |  |  |  |  |  | ↔No significant change in PA level (IPAQ: MET-min/week) from baseline to 12 months between the 2 groups. | |
| Tidhar et al.  (2010) (112) | Israel | 48 | 16 | 32 |  |  | **Aqua Lymphatic Therapy** x 45mins.  1 x per week x 3months. | Low | > 60months post-surgery (Mean) | Water displacement | QOL.  Feeback comments. | ↔ No increase or long term reduction in limb volume. | ↑ Significant improvement in psychological and social dimension of QOL of IG compared to CG.  ↔ No improvement in physical dimension of QOL. | |
|  |  | 56 ±10 | 56.2 ±10.7 | 56.5 ±8.8 |  |  |  |  |  |  |  |  |  |  |
|  |  |  | BMI > 25: 13 (81.3%) | BMI > 25: 24 (75%) |  |  |  |  |  |  |  |  | *↓2 participants were not satisfied with the intervention.* | |
| Schmitz et al.  (2010) (104) | USA | 154 | 77 | 77 |  |  | **RE of upper and lower body.**  3 sets x 10 x 90mins.  2 x per week x 12 months.  AE warm-up | Very light and progressed | > 36months post diagnosis (Mean) | Water displacement. Self-report (Norman Questionnaire). Clinical Examination. | Strength.  IPAQ. | ↔ Less women in the IG experienced ≥5% increase in arm swelling compared to the CG.  ↑ Those with 5 or more nodes removed in the IG experienced less swelling than those in the CG. | ↑ Significantly improved upper body strength of IG compared to CG at 12months. | |
|  |  |  | 54 ±8 | 56 ±8 |  |  |  |  |  |  |  |  | ↔No significant change in PA level (IPAQ: MET-min/week) from baseline to 12 months between the 2 groups. | |
|  |  |  | 27.52 ±5.09 | 28.55 ±6.17 |  |  |  |  |  |  |  |  |  |  |
| Malicka et al.(2011) (90) | Poland | 38 | 23 | 15 |  |  | **Nordic walking** x 60mins. 2 x per week x 8weeks. Warm-up/cool down: upper body stretching, relaxation. | Vigorous | > 60months post-surgery (Mean) | Circumference | Strength. | ↔ no significant difference in lymphoedema in either IG or CG. | ↑ Nordic walking improves function of the upper extremity muscles | |
|  |  | 62.8 ± 6.1 | 63.6 ±6.8 | 63.8 ± 9.2 |  |  |  |  |  |  |  |  |  |  |
|  |  | Reported as height (cm) and body mass (kg) | | | | |  |  |  |  |  |  |  |  |
|  |  | 158.1 ±5.4. 72.3 ±10.9 | 157.2 ±5.5. 72.1 ±11.4 | 159.4 ±10.5 |  |  |  |  |  |  |  |  |  |  |
| Kilbreath et al.  (2012) (84) | Australia | 160 | 81 | 79 |  |  | **RE of upper body** x 8-15 reps each x alternate days.  Passive shoulder stretches x 5-15 min x daily.  8 weeks with follow up at 6months. | Light progressed to vigorous | < 6 weeks post-surgery | Circumference.  BIS.  Self-report (BR23). | QOL.  Strength.  ROM. | ↔ No significant changes in lymphoedema immediately after the intervention and at 6 months between the 2 groups. | ↑Significant increase in shoulder ROM immediate and 6 months post intervention for IG compared to CG  ↑ Significant increase in upper body strength of affected side of IG compared to CG. | |
|  |  |  | 53.5 ±12.1 | 51.6 ±11 |  |  |  |  |  |  |  |  |  |  |
|  |  |  | 26.2 ±5.1 | 26.5 ±4.6 |  |  |  |  |  |  |  |  |  |  |
| Anderson et al.  (2012) (47) | USA | 104 | 52 | 52 |  |  | **AE - walking** x 30mins. **RE: upper and lower body** x 20mins. Compression sleeve. Warmup: aerobic + stretching. 2 x per week x 6 months. Follow up at 1 year. | Moderate progressed to vigorous (AE and RE) | < 12 weeks post-surgery | Water displacement | QOL. Anxiety/  Depression.  6 min walk test. CHAMPS questionnaire. Self-efficacy scale. | ↔ No significant changes in arm volume between groups | ↔ No significant change in QOL between groups.  ↑ Improved physical function of IG at 18months compared to CG.  ↑At 18months participants in the exercise intervention group had significantly higher total metres walked in 6 min walk test. | |
|  |  | 53.6 | *n=* <50: 21 50-<65: 23. 65-<75: 4. >75: 8. | *n=* <50: 23 50-<65: 19. 65-<75: 7. >75: 3. |  |  |  |  |  |  |  |  |  |  |
|  |  |  | *kg/m*² <25:16. 25-29.9: 22. >30: 14. | *kg/m*² <25:14. 25-29.9: 23. >30: 15. |  |  |  |  |  |  |  |  |  |  |
| Jeffs and Wiseman  (2013) (76) | UK | 23 | 11 | 12 |  |  | **Arm exercises** -gravity assisted, isotonic plus usual care: compression hosiery, skin care and general activity/exercise.  10-15mins daily x 12 weeks.  Follow up at 26 weeks. |  |  | Perometry | QOL.  ROM. Function/  disability. Feedback comments. Perceived occupation/  activity level. | ↑ Statistically significant reduction in limb volume at week 26 in the IG but not the CG.  ↔ Difference in relative excess limb volume not statistically significant for the 2 groups at week 26. | ↔ No improvement in QOL or ROM.  ↑ Both groups reported increased activity levels over the 12 weeks as the weather improved. | |
|  |  |  | 66 (51-68) | 64.5 (56-73.5) |  |  |  |  |  |  |  |  |  |  |
|  |  |  | 30.95 (27.22-32.55) | 27.43 (24.97-29.79) |  |  |  |  |  |  |  |  | *↑Comments volunteered suggest that individuals are motivated to continue with exercise because of perceived benefit.* | |
| Johansson et al.  (2013) (79) | USA | 29 | 15 | 14 |  |  | **Aquatic based exercise** x 30min.  Plus compression and massage.  3 x per week x 8weeks. | Light to moderate | > 60months post diagnosis (Median) | Perometry.  BIS. | ROM | ↔ Baseline outcome data for arm volume, lymphedema relative volume, bioimpedance spectroscopy, tissue dielectric constant, and shoulder ROM were similar between groups. | ↑ Maximal muscular strength and muscle endurance increased significantly in RE groups compared to CG.  ↑Significant improvement in physical function domain of QOL for low-load RE compared to CG. | |
|  |  |  | 64 (56-74) | 62 (58-71) |  |  |  |  |  |  |  |  |  |  |
|  |  |  | 30 (21-41.4) | 30.1 (26.9-35.8) |  |  |  |  |  |  |  |  |  |  |
| Cormie et al.  2013b (60) | Australia | 62 | 22 | 21 | 19 |  | **RE of upper and lower body** x 60min.  Low load, High load, control.  Warm-up and cool down of AE and stretches.  2 x per week x 3months. | Moderate or vigorous | > 60 months post diagnosis (Mean) | Circumference. BIS. DXA. Self-report (BR23). | QOL. Strength.  ROM. Function/  disability.  Pain. | ↔ Extent of swelling or symptom severity did not differ between groups across the 3-month intervention. | ↑ Moderate to high intensity RE significantly improves muscle strength, endurance and QOL. | |
|  |  |  | 56.1 ±8.1 | 57 ±10 | 58.6 ±6.7 |  |  |  |  |  |  |  |  |  |
|  |  |  | 30.8 ±6.5 | 30.4 ±5.7 | 28.2 ±6.0 |  |  |  |  |  |  |  |  |  |
| Letellier et al.  (2014) (87) | Canada | 25 | 13 | 12 |  |  | **Aquatic therapy** plus land based exercise with compression sleeve.  60min x 1 per week x 12weeks. |  | > 6 months post-surgery | Circumference.  Water Displacement | QOL.  Strength. Function/  disability.  Pain. | ↔ No statistical difference between groups during the study period.  ↔ No statistical difference within-groups for lymphoedema volume. | ↑ Significant improvement from baseline in hand grip strength in both groups.  ↑ Significant reduction in arm disability in the IG only.  ↑Statistically significant improvement in pain measure for IG. | |
|  |  |  | 56.4 ±9.76 | 53.4 ±9.35 |  |  |  |  |  |  |  |  |  |  |
|  |  |  | 25.7 ±9.76 | 26 ±4.3 |  |  |  |  |  |  |  |  |  |  |
| Loudon et al.  (2014) (89) | Australia | 28 | 12 | 11 |  |  | **Yoga** x 90min initial session x 45min daily x 8 weeks.  Follow up at 12 weeks. |  | > 12months post-surgery (Mean) | Circumference.  BIS.  Tissue Induration. | QOL.  Pain.  Fatigue. Qualitative outcomes reported in subsequent paper. | ↔ No sustained change in tissue induration, BIS at 12weeks.  ↓Significant difference in arm volume between groups at 8-12weeks due to increase in IG. | ↔ No between groups changes in sensations, pain, fatigue, VAS scale, QOL at 12 weeks. | |
|  |  |  | 55.1 ±2.5 | 60.5 ±3.6 |  |  |  |  |  |  |  |  |  |  |
|  |  |  | 29.1 ±4.6 | 25.1 ±4.5 |  |  |  |  |  |  |  |  | *↑Qualitative outcomes identified key themes around well-being, greater awareness of body and improved physical function, improved mental health and social functioning.* | |
| Do et al.  (2015) (64) | South Korea | 46 | 22 | 22 |  |  | **RE of upper body** x 3sets of 10 with 2 min rest between.  Plus CDT.  Warm-up and cool down- stretches.  5 x per week x 8 weeks. | Moderate to somewhat hard |  | Circumference. Self-report (BR23) | QOL. Strength. Function/  disability.  Pain. | ↔ After treatment arm volume showed significant improvement in both IG and CG. | ↑Significant improvements in IG after treatment for physical and cognitive functioning, pain, breast symptoms, strength and function. | |
|  |  |  | 49.7 ±7.05 | 49.6 ±10.35 |  |  |  |  |  |  |  |  |  |  |
|  |  |  | *n=* ≥25: 2. <25:20 | *n=* ≥25: 4. <25:22 |  |  |  |  |  |  |  |  |  |  |
| DiBlasio et al.  (2016) (42) | Italy | 16 |  |  |  |  | **Nordic walking** with/without Isa method x 35mins.  (Isa method uses Isa balls for dynamic exercises attached to Nordic walking poles). Warm-up x 15mins, cool down x 10mins.  3 x per week x 10 lessons. |  |  | Circumference. BIS. |  | ↑ Inclusion of Isa method saw significant reduction in some limb circumference measurements. |  | |
|  |  | 49.09 ±2.24 |  |  |  |  |  |  |  |  |  |  |  |  |
|  |  |  |  |  |  |  |  |  |  |  |  |  |  |  |
| Zhang et al.  (2016) (117) | China | 1000 | 500 | 500 |  |  | **Arm exercises** - remedial physical upper limb exercise. 20-30mins daily x 6months.  Follow up at 1 year. |  | Recruited at diagnosis | Circumference. Clinical examination. | ROM. | ↑ MLD when combined with physical exercise significantly reduced upper limb lymphoedema when compared to physical exercise alone. | ↑ MLD plus physical exercise prevented scar formation and improved function. | |
|  |  |  | no. <50 272 no. ≥50 228 | no. <50 266 no. ≥50 234 |  |  |  |  |  |  |  |  |  |  |
|  |  |  |  |  |  |  |  |  |  |  |  |  |  |  |
| Şener et al.  (2017) (107) | Turkey | 60 | 30 | 30 |  |  | **Clinical Pilates.**  3 x per week x 8weeks. |  |  | Circumference.  Self-report (BR23) | QOL.  Strength.  ROM. Function/  disability.  Pain. Anxiety/  Depression. | ↑ Pilates resulted in a significant reduction in lymphoedema at all points except the axilla for the IG when compared to the CG. | ↑ Pilates resulted in a significant improvement in pain, grip strength, ROM, QOL, anxiety, function when compared to pre-treatment. | |
|  |  |  | 53.17 ±7.66 | 54.03 ±12.57 |  |  |  |  | |  |  |  |  |  |
|  |  |  | 28.53 ±4.51 | 30.35 ±4.99 |  |  |  |  |  |  |  |  |  |  |
| Schmidt et al.  (2017)(103) | Germany | 49 | 21 | 28 |  |  | **Arm crank ergonometer:** strength and endurance training x 60mins.  2 x per week x 12 weeks | Moderate | > 6 months post active treatment (Mean) | Circumference.  BIS.  Self-report (BR23). | QOL.  Strength.  Fatigue. | ↔Decrease in arm circumference was found in both intervention and usual care groups but were not significant between groups. | ↔ Whilst there were improvements in some IG outcomes (lean body mass, skeletal body mass, body fat, physical functioning, general fatigue and physical fatigue), these were not significant between groups. | |
|  |  |  | 61.67 ±10.01 | 53 ±10.7 |  |  |  |  |  |  |  |  |  |  |
|  |  |  | 30.42 ±7.55 | 26.92 ±6.42 |  |  |  |  |  |  |  |  |  |  |
| Donmez and Kapucu  (2017) (65) | Turkey | 52 | 25 | 27 |  |  | **Arm exercises**: Ball squeezing 2 x 3-5mins. Stretching x 15-20min. Aerobic walking X 15 - 20min.  Plus SLD. 2 x per week x 6 weeks. |  |  | Circumference.  Self-report (VAS) | Function/  disability.  Status of performing regular exercise. Walking monitor schedule. | ↔ No significant change in circumference between arms for IG. ↑Statistically significant differences between groups for self-report symptoms. | ↑ Mean DASH scores were significantly significant, over time, between groups. 40% of IG and 33.3% CG exercised regularly at baseline. | |
|  |  |  | 48.6 ±8.3 | 49.5 ±11.9 |  |  |  |  |  |  |  |  |  |  |
|  |  |  | 20-24.9 *n=9.* 25-29.9 *n=16* | 20-24.9 *n=12.* 25-29.9 *n=15* |  |  |  |  |  |  |  |  |  |  |
| Ammitzbøll et al.  (2019) (46) | Denmark | 158 | 82 | 76 |  |  | **RE of upper and lower body plus core** x 10-20reps x 1-4 weeks of 2-3 sets. Week 5 -week 50 x 3 sets.  Warm-up: AE. 3 x per week x 20 weeks (supervised).  Then 3 x per week from week 20 to week 50 (self-administered). | Moderate | < 6 weeks post-surgery. (During adjuvant treatment). | Water displacement. Self-report (NRS). Clinical examination. | Strength.  ROM. Questionnaire for PA before diagnosis. | ↔ no significant inter limb volume differences between groups at 12 months. | ↔ No difference to between groups isometric muscle strength or ROM at 12 months.  ↑ Significantly greater increase in strength in the IG compared to CG for leg press, elbow flexion, contralateral shoulder abduction. Level of PA before diagnosis was high. | |
|  |  |  | 53 ±10 | 52 ±10 |  |  |  |  |  |  |  |  |  |  |
|  |  |  | 26.5 ±6 | 26.6 ±5 |  |  |  |  |  |  |  |  |  |  |
| Bloomquist et al.  (2019) (50) | Denmark | 153 | 75 | 78 |  |  | **RE high intenisty of upper and lower body** x 2-3 sets of 8-12 reps. Warm-up- AE 10-15mins.  At 6 weeks plus **AE (All sports e.g. ballgames, dancing, static bikes).** 2-3 x per week x 12 weeks. | RE Vigorous to near maximal-maximal. AE: Vigorous to near maximal. | Undergoing active chemotherapy | BIS.  DXA.  Self-report (NRS. BR23). | QOL.  Strength.  Pain. Qualitative interviews. | ↔ Similar point prevalent cases of lymphoedema between groups. Similar between group L-Dex scores, perceptions of heaviness, swelling and tightness post-intervention. | ↑ Significantly greater improvement in upper extremity strength at 6 and 12 weeks in the high intensity group compared to low intensity group.  ↔ No between groups difference for QOL. Outcomes of qualitative interview not reported on. | |
|  |  | 51.7 ±9.4 | 51.5 ±9.6 | 52 ±9.3 |  |  |  |  |  |  |  |  |  |  |
|  |  | 26.1 ±5.1 | 26.2 ±5.3 | 26.0 ±4.9 |  |  |  |  |  |  |  |  |  |  |
| Odynets et al.  (2019) (97) | Ukraine | 68 | 34 | 34 |  |  | **Aquatic exercise vs Pilates** exercise x 1 hour.  3 times per week x 12 weeks. | Individualised | < 6 months post-surgery (Mean) | Circumference | Strength.  ROM. | ↑ Aqua exercise group significant changes for the forearm and hand areas compared to Pilates group. | ↑ Significant improvement in ROM and strength of both groups. | |
|  |  |  | 57.44 ±2.16 | 57.99 ±2.24 |  |  |  |  |  |  |  |  |  |  |
|  |  |  | 25.92 ±0.42 | 26.01 ±0.81 |  |  |  |  |  |  |  |  |  |  |
| Schmitz et al.  (2019) (106) | USA | 351 | 87 | 87 | 87 | 90 | **RE of upper and lower body** x taught as 90mins, then 2-3 x 10 reps.  **AE walking** x 90 - 180mins per week. Compression sleeve. Warmup/ cool down: stretching, core, strength.  X 1 year | Light and progressed | > 60months post diagnosis (Mean) | Perometry.  Self-report (Norman questionnaire). Clinical examination. | QOL.  Strength. Function.  Fatigue.  Pain.  Modifiable PA questionnaire. | ↑Perometry -Individual limb decreases were larger for both affected and unaffected limbs in weight loss and combine intervention groups vs CG across 12 months.  ↔ No between group differences for lymphoedema exacerbations, cellulitis, clinical evaluation or self-report at baseline/12 months | ↑Significant weight loss for combined and weight loss groups vs CG. ↑Strength of upper and lower body and aerobic fitness increased more in all groups compared to  CG. | |
|  |  |  | 59.1 ±8.1 | 59.4 ±9.2 | 60.0 ±9 | 59.0 ±8.5 |  |  |  |  |  |  |  |  |
|  |  |  | 34.0 ±6.2 | 33.8 ±5.6 | 34.2 ±6.3 | 34.0 ±5.7 |  |  |  |  |  |  |  |  |
| Pasyar et al.  (2019) (100) | Iran | 40 | 20 | 20 |  |  | **Yoga** x 3 x per week x 8 weeks |  |  | Water displacement | QOL. | ↔ No significant difference in lymphoedema volume between groups at 4 and 8 weeks. | ↑ At 8 weeks significant difference in physical and emotional functioning of QOL in IG vs CG but no difference for other sub-scales. | |
|  |  |  | 51.6 ±10.46 | 51.8 ±11.4 |  |  |  |  |  |  |  |  |  |  |
|  |  |  | 29.33 ±4.07 | 30.63 ±5.28 |  |  |  |  |  |  |  |  |  |  |
| Kilbreath et al.  (2020) (82) | Australia | 89 | 41 | 47 |  |  | **RE or upper and lower body plus core** x 30min.  **AE of stationary bike, treadmill and cross-trainer** (1 hour total). Warm-up- stretching.  3 x per week x 12 weeks. | Moderate to maximum |  | BIS.  US.  Self-report (Lymphoedema Symptom Intensity and Distress Survey and BR23) | QOL.  Strength. Cardiorespiratory fitness. | ↔No significant change in dermal thickness between groups. BIS. ↔Only significant between groups change with BIS was in the breast. | ↑Significant improvement in breast symptoms in the QOL measure for IG vs CG. ↑Increased upper and lower limb strength in IG than in CG.  ↔No significant difference between groups for intensity and distress. | |
|  |  |  | 53.7 ±10.4 | 59.5 ±8.0 |  |  |  |  |  |  |  |  |  |  |
|  |  |  | 28.1 ±5.5 | 30.4 ±5.9 |  |  |  |  |  |  |  |  |  |  |
| Omar et al.  (2020) (98) | Egypt | 70 | 30 | 30 |  |  | **RE of upper body**. Sets x 12. Plus or minus compression. Warmup/cool down of mobility and stretching x 15min. 3 x per week x 8 weeks.  Follow up at 12 weeks. | Moderate | > 60months post-surgery (Mean) | Circumference.  Self-report (VAS). | ROM.  Function/  disability. | ↔Whilst there were reductions in excess limb volume and relative volume in both groups, there were no statistically significant changes in relative volume between groups at the end of treatment or follow-up.  ↑ Self-reported symptoms reduced in both groups but were not statistically significant between groups i.e. if compression was worn or not. | ↑ Increased shoulder ROM in both groups but not statistically significant. ↔No difference in function between groups at the end of treatment. | |
|  |  |  | 53.78 ±2.99 | 52.62 ±2.92 |  |  |  |  |  |  |  |  |  |  |
|  |  |  | 30.23 ±4.81 | 29.85 ±4.75 |  |  |  |  |  |  |  |  |  |  |
| Naughton et al.  (2021) (96) | USA | 547 | 309 | 238 |  |  | **Arm exercises**: stretching, strengthening and breathing plus education x 15mins. Daily x 18months. |  | Recruited prior to surgery or neoadjuvant chemotherapy | Circumference.  Self-report.  Clinical examination. | QOL.  ROM.  Pain. | ↔Presence of lymphoedema at 18months was only borderline significantly related to health related QOL. | ↑Positive increase in emotional functioning beginning at 6 months and continuing to the end of the trial.  ↓Physical and social functioning scores did not reach their pre-surgery levels by 18months post-surgery.  ↔Outcomes of questionnaire on lymphoedema knowledge, self-efficacy, body image and fear of recurrence not reported. | |
|  |  | 57.6 ±11.3 | 57.4 ±11.2 | 58 ±11.5 |  |  |  |  |  |  |  |  |  |  |
|  |  | 28.2 ±5.7 | 28.0 ±5.4 | 28.4 ±6.0 |  |  |  |  |  |  |  |  |  |  |
| Bruce et al.  (2021) (56) | UK | 382 | 191 | 191 |  |  | **Arm exercise** post operatively progressed at 1 month post-operatively to a combination of **RE of upper body** x 30-60mins plus 150min **AE per week.**  3-6 Therapy led sessions followed by 2 x per day x 1 year. | RE: Progress to heavy. AE: Moderate to hard | Recruited at the time of diagnosis | Self-report (LBCQ) | QOL.  Function/  disability.  Pain. | ↔No significant difference in self-report lymphoedema symptoms between groups at 6 weeks, 6 months or 12 months. | ↑Significant improvement in pain, QOL, function at 12 months for IG vs CG.  ↔ No effect on PA rates at 1 year | |
|  |  | 58.1 ±12.1 | 58.4 ±12.2 | 57.8 ±12.0 |  |  |  |  |  |  |  |  |  |  |
|  |  | 30.2 ±7 | 29.9 ±6.9 | 30.6 ±7.2 |  |  |  |  |  |  |  |  |  |  |
| Bloomquist et al.  (2021) (52) | Denmark | 68 | 46 | 22 |  |  | **Football**: warmup and football drills x 60mins. 2 x per week x 52weeks | Light to near maximal | > 36months post-surgery (Mean) | BIS.  DXA.  Self-report (BR23) | QOL. Function/  disability. | ↔No significant difference between groups, in arm volume % difference at 6 or 12 months, in L-Dex score at 12 months, and in breast/arm symptoms at 6 and 12 months. These results were consistent with further analysis for those with 5 or more lymph nodes removed. | ↔No differences in upper extremity function at 6 and 12 months between groups. ↑Participants continued to train together after the intervention | |
|  |  |  | 47.4 ±9.4 | 50.0 ±9.3 |  |  |  |  |  |  |  |  |  |  |
|  |  |  | 25.5 ±4.9 | 26.4 ±4.6 |  |  |  |  |  |  |  |  |  |  |
| Ali et al.  (2021) (45) | Egypt | 50 | 25 | 25 |  |  | **Aquatic therapy**. Warmup x 10mins stretching. Main: 40-45mins. 3 x per week x 8 weeks | Moderate and progressed | > 24 months post-surgery (Mean ) | Circumference (limb volume) | ROM.  Pain. | ↑Significant difference in limb volume post treatment for IG vs CG.  ↑Significant decrease in limb volume for both IG and CG from pre to post treatment. | ↑Significant increase in shoulder flexion and abduction ROM and decrease in pain score post treatment for IG vs CG.  ↑Both groups showed significant increase in shoulder flexion/abduction ROM post treatment vs pretreatment. | |
|  |  |  | 51.36 ±9.15 | 49.84 ±8.57 |  |  |  |  |  |  |  |  |  |  |
|  |  |  | 29.97 ±3.36 | 29.78 ±4.0 |  |  |  |  |  |  |  |  |  |  |
| Lin et al.  (2022) (88) | China | 192 | 48 | 49 | 47 | 48 | **Joint mobility exercise of upper body and back x**15mins x 3 x per day PLUS intensive follow up ;  PLUS **RE of upper and lower body x** 5 mins x 8 exercises x 2-3 x per day;  OR **AE brisk walk** x 35mins x 5 x week. 6 months.  Follow up at 9 months and 12 months. | RE: Moderate and progressed. AE: Moderate/ vigorous | < 6 weeks post-surgery | Circumference (limb volume) | QOL.  Pain. | ↑Significant difference between joint mobility exercise plus intensive follow-up plus either RE or AE compared to joint mobility and intensive follow up alone 6 months post intervention. | According to group effect: ↑Joint mobility exercise plus RE plus intensive follow-up group had the best effect on improving QOL.  ↑Joint mobility exercise plus AE plus intensive follow-up group had the best effect in improving pain. | |
|  |  | 51.61 ±30.71 | 49.58 ±10.91 | 47.37 ±9.99 | 49.38 ±9.51 | 51.69 ±10.14 |  |  |  |  |  |  |  |  |
|  |  | 23.36 ±3.60 | 22.73 ± 2.67 | 22.92 ± 3.27 | 23.90 ± 4.37 | 23.90 ±3.85 |  |  |  |  |  |  |  |  |
| Naczk et al.  (2022) (43) | Poland | 24 |  |  |  |  | **RE - Inertial - eccentric exercise of upper body** x 12-14 reps. Warm-up of shoulder exercises. 2 x per week x 6weeks. | Vigorous | > 60months post-surgery (Mean) | BIS | Strength. Function. | ↔Percentage changes did not differ significantly between groups. | ↑ Percentage increases in strength were significantly more in the IG than the CG. ↔ No significant changes in function between groups. | |
|  |  | 66.2 ±10.6 |  |  |  |  |  |  |  |  |  |  |  |  |
|  |  | Body mass: 72.7 ±10.6 Height: 159 ±5.2cm | | | |  |  |  |  |  |  |  |  |  |
| Balaji and Senthil(2024) (49) | India | 17 |  |  |  |  | **RE of upper bod**y x 3 sets of 15.15.15 with theraband +MLD x 45min.  3 x per week x 8 weeks. Follow up at 12 weeks. | Light |  | Circumference | QOL. | ↑Significant decrease in arm circumference in IG vs CG post-test. | ↑Significant increase in QOL in IG vs CG post-test. | |
|  |  | 30-60 |  |  |  |  |  |  |  |  |  |  |  |  |
|  |  |  |  |  |  |  |  |  |  |  |  |  |  |  |
| Venugopalan et al.  (2024) (115) | India | 10 |  |  |  |  | **RE of upper body** plus routine post operative ROM exercises x 12 weeks. |  | < 6 weeks post-surgery | Circumference.  Water displacement. | QOL.  Strength.  ROM. Pain/disability. | ↑Mean increase of arm circumference in IG was significantly more than in CG.  ↔No significant difference between groups for limb volume. | ↔No significant change in ROM (except external rotation) or strength between groups.  ↑Significant improvement of pain/disability in IG vs CG. ↑ QOL improved more in IG than CG. | |
|  |  | 49.9 ±8.6 |  |  |  |  |  |  |  |  |  |  |  |  |
|  |  | 28.4 ±3.9 |  |  |  |  |  |  |  |  |  |  |  |  |
| Harvie et al.  (2024) (74) | UK | 57 | 12 | 16 | 17 | 12 | **RE of upper body** plus supervised **moderate intensity AE** (150min per week) and weight control (diet) x 12 weeks. | AE: Moderate | > 60 months post diagnosis (Median) | Perometry. Self-report (FACT-B+4). BIS. |  | ↑Small reduction in limb volume in supervised IG.  ↑Large scale changes in BIS. ↔ No change in self report symptoms. ↔No change in perometer. | ↑Reduced body weight and body fat in IGs vs CG. | |
|  |  | 62.6 ±8.8 | 63.0 ±8.9 | 61.9 ±9.4 | 58.2 ±9.2 | 63.8 ±6.2 |  |  |  |  |  |  |  |  |
|  |  | 29.8 ±4.5 | 30.3 ±4.5 | 30.08 ±5.0 | 29.5 ±3.5 | 28.9 ±4.2 |  |  |  |  |  |  |  |  |
| Esteban-Simon et al.  (2024) (67) | Spain | 60 | 32 | 28 |  |  | **RE upper and lower body** + **home based AE** (10,000 steps per day). Plus Warm up: AE, stretch, mobility x 15min. 2 x per week x 12 weeks. | Light progressed to vigorous | > 36 months post active treatment (Median) | Perometry | QOL.  Strength. Function/  disability. | ↔No difference between groups arm volume or in sub-analysis for treatment type or number of nodes removed. | ↔No difference between arm shoulder disabilities or in sub-analysis for treatment type or number of nodes removed.  ↔Steps per day similar for both IG and CG. | |
|  |  |  | 52.6 ±8.8 | 52.0 ±9.4 |  |  |  |  |  |  |  |  |  |  |
|  |  |  | 27.4 ±4.2 | 26.3 ±5.3 |  |  |  |  |  |  |  |  |  |  |
| Nakamoto et al.  (2024) (95) | Japan | 356 | 115 | 104 | 111 |  | **Curves programme of AE and RE of upper and lower body and abdominals** x 30mins. 3 x per week x 16 weeks. Follow up at 36 weeks post intervention. |  | < 6 weeks post-surgery (Mean) | Circumference | Recreational PA (reported in associated paper) | ↔No significant differences on the effect of the intervention at 12months between IG and CG, and between education and CG.  ↔No significant difference in the incidence of lymphoedema at each time point between groups, except baseline. change in lymphoedema risk.  ↑Axillary dissection and obesity significantly increased the risk of lymphoedema but ↔ there were no significant differences in the effect of the interventions on lymphoedema risk. | ↔No significant differences in recreational PA between groups at 1 year. | |
|  |  |  | 54.3 ±10.8 | 55.4 ±10.4 | 55.2 ±11.3 |  |  |  |  |  |  |  | ↑some significant improvements in body composition including BMI, body weight and body fat percentage, were observed in the education and exercise intervention groups. | |
|  |  |  | 23.0 ±3.1 | 23.3 ±3.4 | 23.2 ±3.8 |  |  |  |  |  |  |  |  |  |
| Giron et al.  (2025) (71) | Brazil | 79 | 29 | 26 | 24 |  | **RE of upper body**, 3 x 10reps of each and **stretching** (kinesiotherapy exercises) x 30mins.  1 x per week x 10weeks |  | > 24 months since surgery (Mean) | Perometry | QOL. Strength. | ↔No statistically different changes between groups. | ↔Despite statistically different changes within groups for strength and QOL there were no statistically significant changes between groups. | |
|  |  |  | 52.8 ±10.4 | 53.65 ±9.3 | 52.1 ±9.3 |  |  |  |  |  |  |  |  |  |
|  |  |  | 29.1 ±5.2 | 28.2 ±5.8 | 30.6 ±5.9 |  |  |  |  |  |  |  |  |  |
| Maraş Baydogan et al.  (2025) (91) | Turkey | 40 | 20 | 20 |  |  | **Arm exercises** (mobility) x 10-30min and muscle relaxation training x 30min.  3 -7 sessions daily x 3months. |  | Recruited at time of surgery | Circumference.  Self-report (self-care scale for BCRL). Symptom warning model for lymphoedema. |  | ↔Statistically significant changes in arm measurements between groups at 1 and 3 months but not between pre-op and 3 months. ↑5% of IG developed high risk of lymphoedema vs 30% CG in first 1-3months.  ↑Significant improvement in self-care of IG post intervention. |  | |
|  |  |  | 49.10 ±8.71 | 49.25 ±10.4 |  |  |  |  |  |  |  |  |  | |
|  |  |  |  |  |  |  |  |  |  |  |  |  |  |  |
| Ramirez-Parada et al.  (2025) (101) | Chile | 116 | 61 | 55 |  |  | **RE of upper and lower body** x 10 exercises x 40min. Warm-up/cool-down x 5mins. 2 x per week x 12 weeks. Follow up at 36 weeks. | Moderate | Recruited prior to starting chemotherapy | Perometry.  Self-report (BR23) | QOL.  Strength.  6 min walk test. Smart bracelet for daily steps. | ↑The IG has a significant decrease in arm volume from baseline to 36weeks vs the CG.  ↓Presence of AWS had a significant effect on increasing arm volume of both groups. ↔BMI had no effect. | ↑ Significant increase in grip strength in both groups from baseline to 36weeks. From QOL, both groups were associated with ↓significantly increased systemic therapy side effects but ↑ reduced arm symptoms over time.  ↑Significant increase in functional capacity, daily steps and reduction in body weight in intervention group. | |
|  |  | 54.08 ±9.51 | 53.3 ±9.41 | 54.9 ±9.64 |  |  |  |  |  |  |  |  |  |  |
|  |  | 30.3 ±5.15 | 29.2 ±5.07 | 31.0 ±5.12 |  |  |  |  |  |  |  |  |  |  |
|  |  |  |  |  |  |  |  |  |  |  |  |  |  | |
| Zhang et al.  (2025) (118) | China | 110 | 36 | 37 | 36 |  | **RE of upper body** x 15-20min. Warmup: Deep breathing and mobility x 7min. Cool down: Stretching x 5-10min. ?1-2 x per day. 12 weeks. Follow up at 3 months. | Light progressed to vigorous | Recruited at time of surgery | BIS | Strength. | ↔Moderate to high intensity RE had significantly lower extra-cellular water than CG or low load group post-intervention and at 3 month follow-up. However, at 6 months follow-up the high intensity group had significantly higher water content than the low intensity group, but this was still lower than the CG. | ↑Significant increase in grip strength in both IG at 3 and 6 months in comparison to the CG but not between IGs. | |
|  |  |  | 49.11 ±9.35 | 46.73 ±10.98 | 51.54 ±4.57 |  |  |  |  |  |  |  |  |  |
|  |  |  | 25.38 ±3.58 | 26.11.38 ±3.66 | 25.86 ±3.88 |  |  |  |  |  |  |  |  |  |
| Single group (n=15) | | | | | | | | | | | | | |  |
| Lane et al.  (2005) (86) | Canada | 16 |  |  |  |  | **AE (of choice)** x 30-45mins x 3x per week. **RE of upper body x** 2 x 10reps.  Warm-up of AE x 5-7min.  Cool-down - stretching. 3 x per week.  **Dragon boat training** x 15min warmup and 90min paddling.  2 x per week. 20 weeks. | RE: light and progressed. AE: moderate | > 12 months post diagnosis (Median) | Circumference.  Water displacement | Strength. | ↔Both arms showed a significant increase in volume and circumference. | ↑Both arms strength increased significantly. | |
|  |  | 52.4 ±6.8 |  |  |  |  |  |  |  |  |  |  |  |  |
|  |  | 24.1 ±2.8 |  |  |  |  |  |  |  |  |  |  |  |  |
| Johansson and Piller  (2007) (77) | Australia | 18 |  |  |  |  | **RE of upper body** x 10reps x 5 exercises. 5 sessions. | Light and progressed to strenuous |  | BIS.  Water displacement. Self-report (VAS). | Rate of perceived exertion. | ↑Statistically significant reduction in self-reported heaviness and tension 24hours after training.  ↔ Significant increase in arm volume immediately after exercise but no change after 24hrs. |  | |
|  |  | 58 ±11 |  |  |  |  |  |  |  |  |  |  |  |  |
|  |  |  |  |  |  |  |  |  |  |  |  |  |  |  |
| Jönsson and Johansson  (2009) (80) | Sweden | 26 |  |  |  |  | **Pole walking** x 1 session | Light to Moderate | > 60 months post-surgery (Mean) | Water displacement . Self-report (VAS) |  | ↔ No significant difference in total arm volume (TAV) in the oedema arm immediately after pole walking or 24 hours later compared to before walking. |  | |
|  |  | 58.4 ±6.4 |  |  |  |  |  |  |  |  |  |  |  | |
|  |  | 25.2 ±3.2 |  |  |  |  |  |  |  |  |  |  |  | |
| Gautam et al.  (2011) (70) | India | 32 |  |  |  |  | **RE of upper body** . 1-2reps.  Increasing reps from 8-15.  Stretching and deep breathing.  Warm-up ROM X 5mins.  5 x per week x 8 weeks. | Moderate | > 24 months post-surgery (Mean) | Circumference.  Water displacement | QOL.  Feedback comments. | ↑Statistically significant difference in the affected arm upper limb volume at all measurements except metacarpophalangeal joint at 8weeks | ↑Statistically significant difference was found in QoL post intervention. | |
|  |  | 46.56 ±6.98 |  |  |  |  |  |  |  |  |  |  | *↑Participants reported more confidence using affected limb and were less reminded of having lymphoedema.* | |
|  |  |  |  |  |  |  |  |  |  |  |  |  |  |  |
| Stan et al.  (2012) (111) | USA | 15 |  |  |  |  | **Pilates** x 45min. 2x per week for weeks 1-4. 3 x per week for weeks 5-8.  4 x per week for weeks 9-12.  12 weeks total. |  | < 6 months post-surgery (Mean) | Perometry. Self-report (Norman questionnaire) | QOL.  ROM.  Mood. Questionnaire intervention evaluation. | ↓Limb volume of affected arm increased relative to the unaffected arm over 12 weeks | ↑Overall shoulder mobility, mood and body image improved but not elements were statistically significant.  ↑ Statistically and significant increase in all QOL scores except emotional well-being. | |
|  |  | 49 ±9 |  |  |  |  |  |  |  |  |  |  |  |  |
|  |  |  |  |  |  |  |  |  |  |  |  |  | *↑All answered positively to the Pilates exercises* | |
| Jönsson and Johansson  (2014) (81) | Sweden | 35 | 23 |  |  |  | **Pole Walking x 30-60min**.  Warmup: 10min. 3-5 times per week x 10 weeks. | Vigorous | > 60 months post-surgery (Mean) | Water displacement. Self-report (VAS). | Function/  Disability. Exercise questionnaire. C  V fitness. | ↑Significant reduction of arm volume at post-intervention.  ↑Significant improvement in the rating of tightness but not heaviness post intervention. | ↑Significant decrease in HR and DASH post intervention.  ↔No change in BMI. | |
|  |  |  | 60.4 ±8.3 |  |  |  |  |  |  |  |  |  |  |  |
|  |  |  | 26.6 ±4.3 |  |  |  |  |  |  |  |  |  | *↑ all participants reported improved well-being.* | |
| Fisher et al.  (2014) (68) | USA | 6 |  |  |  |  | **Yoga** x 60min.  3 x per week x 8 weeks. | Low | > 60 months post diagnosis (Mean) | Water displacement | QOL. Strength. Function/  disability. | ↑Significant decrease in arm volume from pre to post intervention. | ↔ no significant change in QOL, grip strength or arm function from pre-post intervention. | |
|  |  | 57 ±7 |  |  |  |  |  |  |  |  |  |  |  |  |
|  |  | 27.8 ±5.6 |  |  |  |  |  |  |  |  |  |  |  |  |
| Yen-Ting Lai et al.  (2017) (116) | Taiwan | 15 |  |  |  |  | **Yoga** x 60min.  3 x per week x 12 weeks. |  |  | Water displacement. Self-report (VAS). |  | ↔ no change in lymphoedema of the affected arm compared to the unaffected arm post intervention. |  | |
|  |  | 42-72 |  |  |  |  |  |  |  |  |  |  |  |  |
|  |  |  |  |  |  |  |  |  |  |  |  |  |  |  |
| Godoy et al.  (2017) (72) | Brazil | 30 |  |  |  |  | **Arm bike exercises 12 mins with 3 mins rest** x 60mins. Compression sleeve.  1 session. |  |  | Water displacement. |  | ↑ Significant reduction in arm volume from pre to post intervention. |  | |
|  |  | 61.5 ±37 |  |  |  |  |  |  |  |  |  |  |  |  |
|  |  |  |  |  |  |  |  |  |  |  |  |  |  |  |
| Simonavice et al.  (2017) (108) | USA | 27 |  |  |  |  | **RE upper and lower body** x 2 sets x 8-12 reps for 60mins.  Warm up of AE (walking). Compression.  2 x per week x 6months. | Moderate to Vigorous | > 60 months post diagnosis (Mean) | Circumference | QOL.  Strength. Function. | ↔ No significant differences in arm circumference post intervention | ↑Significant improvement in function from baseline but not QoL.  ↔ no difference in body composition or bone mineral density | |
|  |  | 64 ±7 |  |  |  |  |  |  |  |  |  |  |  |  |
|  |  | 27.7 ±5.5 |  |  |  |  |  |  |  |  |  |  |  |  |
| Ammitzboll et al.  (2017) (40) | Denmark | 8 |  |  |  |  | **RE upper and lower body plus core** x 40mins.  Warm up: Mobility x 10-15min. 60min total. 3 x per week x 20weeks.  Followed by weeks 20-50 at home. | Maximal to submaximal | < 6 weeks post-surgery (Mean) | Water displacement. Self-report (NRS) | QOL.  Strength.  BMI.  ROM.  Pain. Anxiety/  Depression. Fatigue. Questionnaire intervention evaluation.  IPAQ. | ↓ 1/8 participants showed had subjective and objective lymphoedema symptoms at 50weeks. ↑ROM recovered in all participants except 1. | ↑ Increased strength post intervention and minimal difference between arms in all measurements of strength at 50-week follow-up. IPQA reported at baseline. | |
|  |  | 57.4 (±7.5) |  |  |  |  |  |  |  |  |  |  |  |  |
|  |  | 24 ±2.8 |  |  |  |  |  |  |  |  |  |  | *Participants wished for individual treatment at the community centre.* | |
| Mazor et al.  (2018) (92) | USA | 21 |  |  |  |  | **Yoga** x 60min.  2 x per week x 8 weeks. |  | > 24 months post diagnosis (Mean) | Circumference | Strength.  ROM. | ↔No pre-post intervention change in limb volume in either arm. | ↔No significant changes in shoulder, elbow or wrist ROM of the affected limb. | |
|  |  | 52.0 ±9.1 | |  |  |  |  |  |  |  |  |  |  |  |
|  |  | 24.8 ±5.1 | |  |  |  |  |  |  |  |  |  |  |  |
| Salacinski et al.  (2021) (41) | USA | 10 |  |  |  |  | **Aquatic therapy**/water aerobics - whole body work out with RE x 60min.  2 x per week x 15weeks | Moderate to High-moderate | > 60 months post diagnosis (Mean) | BIS |  | ↔No significant changes in body composition including extracellular water across the 12 weeks |  | |
|  |  | 59.40 ± 8.22 |  |  |  |  |  |  |  |  |  |  |  |  |
|  |  |  |  |  |  |  |  |  |  |  |  |  |  |  |
| Carretti et al.  (2022) (58) | Italy | 16 |  |  |  |  | **Upper body ROM** (APA - adapted physical therapy) X 50min.  2 x per week x 8weeks |  |  | Circumference.  US | QOL.  Pain. Anxiety/ Depression. Questionnaire intervention evaluation. | ↔No significant reduction in limb volume. | ↑Significant improvement in wrist mobility, pain, QOL. Trend for improvement in anxiety. | |
|  |  | 65.2±8.6 | |  |  |  |  |  |  |  |  |  | *↑Feedback that it was a positive and effective experience.* | |
|  |  | 32.04 ±3.2 |  |  |  |  |  |  |  |  |  |  |  |  |
| Pardo-Hernandez et al.  (2024) (99) | Mexico | 23 |  |  |  |  | **RE of upper body** plus AE warmup (30mins on static bikes). 2 x per week x 10weeks. | Light progressed to vigorous | < 60 months since surgery | Circumference | Strength. | ↔ no significant differences in upper limb volumes post intervention | ↑ significant differences in muscle mass, lean mass, fat mass and body water post intervention | |
|  |  | 55.7 ± 9.508 |  |  |  |  |  |  |  |  |  |  |  |  |
|  |  | 27.72 ±3.64 |  |  |  |  |  |  |  |  |  |  |  |  |
| Randomised cross-over (n=8) | | | | | | | | | | | | | |  |
| Johannsson et al.  (2005) (78) | Sweden | 31 |  |  |  |  | **RE- upper body** with free weights and with compression.  5 days. | Very light |  | Water displacement. BIS.  Self-report (VAS). | PA questionnaire (Developed by Johannsson et al. 2002). Perceived exertion. | ↓ Significant increase in total arm volume was found immediately after exercise with or without sleeve, this increase was lost by 24hrs.  ↔ No changes in VAS or BIS the day after exercise. | PA reported at baseline. Average of 4.6 ±3.6 took weekly regular PA. ↔Most rated the exercises as very light. | |
|  |  | 55.3 ±7.3 |  |  |  |  |  |  |  |  |  |  |  |  |
|  |  | 25.9 ±3.2 |  |  |  |  |  |  |  |  |  |  |  |  |
| Bracha and Katz-Leurer  (2012) (55) | Israel | 16 |  |  |  |  | **Arm exercises.** Compression.  1 day per week x 3 weeks. |  | > 48 months post diagnosis (Median) | Circumference |  | ↑ Proximal arm muscle exercise caused immediate significant effect on arm volumes. |  | |
|  |  | 62 ±6 |  |  |  |  |  |  |  |  |  |  |  | |
|  |  |  |  |  |  |  |  |  |  |  |  |  |  |  |
| Cormie et al.  (2013a) (59) | Australia | 17 |  |  |  |  | **RE of upper body**. 2 sets of 5 exercises. Heavy load x6-8reps. Low load x15-20reps.  1 off intervention over 2 week period. | Very light. Near maximal/maximal | > 60 months post diagnosis (Mean) | Circumference.  BIS.  DXA.  Self-report (VAS). | Pain.  Tolerance of exercise sessions. | ↔ No significant differences were observed in lymphoedema symptoms or volume, circumference of the affected arm across most of the time  points examined. | ↔ No significant differences in the severity of pain, heaviness, or tightness were observed across all time points examined. ↑Exercise sessions tolerated well. | |
|  |  | 61.2 ±9.1 |  |  |  |  |  |  |  |  |  |  |  |  |
|  |  | 31.5 ±6.9 |  |  |  |  |  |  |  |  |  |  |  |  |
| Singh et al.  (2015) (109) | Australia | 25 |  |  |  |  | **RE of upper body** with compression x 3sets x6. Warm up: AE x 5-10min. 2 sessions. | Moderate | > 60 months post diagnosis (Mean) | Circumference.  BIS.  Self-report (VAS) | Rate of perceived exertion. | ↑ Transient but statistically significant decrease in lymphoedema (with compression).  ↔ no change to lymphoedema outcomes without compression. | 76% Currently physically active as per guidelines at baseline. | |
|  |  | 61.5 ±9.2 |  |  |  |  |  |  |  |  |  |  |  |  |
|  |  | 30.9 ±5.7 |  |  |  |  |  |  |  |  |  |  |  |  |
| Cormie et al.  (2016) (61) | Australia | 21 |  |  |  |  | **RE of upper body** x 6 exercises.  2 x per week x 2 weeks. Then 1 x per week x 3 weeks. | Moderate; Vigorous; Near Max | > 60 months post diagnosis (Mean) | Circumference.  BIS.  Self-report (VAS) | Perceived tolerance.  Rate of perceived exertion. | ↑BIS and circumference tended to decrease at 24hours after exercise in all 3 conditions.  ↑Tightness symptoms decreased significantly in the affected arm 24hours after high load RE. | ↔Exercises rated between light and somewhat hard. No significant effect on inflammatory markers. | |
|  |  | 61.5 ±10.1 |  |  |  |  |  |  |  |  |  |  |  |  |
|  |  | Total: Normal *n=* 2; Overweight *n=* 8; Obese *n=* 12 | | | | |  |  |  |  |  |  |  |  |
| Bloomquist et al.  (2018) (53) | Denmark | 21 |  |  |  |  | **RE of upper body** x 4 exercises. Low load x 15-20reps. High load: 5-8 reps.  Warm up: AE x 10min. 2 sessions with 7 day wash out period over 1 week. | Moderate; Vigorous | Undergoing active chemotherapy | BIS.  DXA.  Self-report (NRS). Clinical examination |  | ↔ For L-Dex and interarm volume outcomes, individual responses to resistance exercise sessions varied with no apparent group trend observed |  | |
|  |  | 45.3 ±9.2 |  |  |  |  |  |  |  |  |  |  |  | |
|  |  | 25.3 ±4.7 |  |  |  |  |  |  |  |  |  |  |  |  |
| Deacon et al.  (2019) (63) | Australia | 18 |  |  |  |  | **Aquatic therapy** x 50mins.  1 x per week x 2 weeks. | Slow, relaxing, gentle |  | Water displacement. BIS | Questionnaire intervention evaluation. | ↔ Immediate reduction in arm volume subsided after 1 hour. | *↓Participants found exercising at slow speed cold. More active classes preferred.* | |
|  |  | 52-81 |  |  |  |  |  |  |  |  |  |  |  |  |
|  |  | < 25: *n=*6; >25-<40: *n=*7; ≥40: *n* =5 | | | | |  |  |  |  |  |  |  |  |
| Guloren et al.  (2023) (73) | Turkey | 34 |  |  |  |  | **Arm exercises** x 10 reps of each exs x 1set (mobilisation, ROM, stretching). Compression.  3-5days |  | > 60 months post-surgery (Mean) | Circumference.  BIS.  Self-report (VAS) |  | ↑ Daily (performed within 24hrs) remedial exercises with compression bandaging provided a significant reduction in severity of lymphoedema and lymphoedema related symptoms. |  | |
|  |  | 59.8 ±8.78 |  |  |  |  |  |  |  |  |  |  |  | |
|  |  | 29.6 ±4.21 |  |  |  |  |  |  |  |  |  |  |  | |
| Non-randomised controlled trials (n=8) | | | | | | | | | | | | | |  |
| Moseley et al.  (2005) (94) | Australia | 52 | 24 | 28 |  |  | **Arm exercises** - mobility and deep breathing. X 10mins. Daily x 1 week. Follow up 1 month. | Gentle |  | Perometry, BIS, Tonometry. Self-report (VAS). |  | ↑Significant reduction in lymphoedema volume and % immediately after the intervention and sustained for 24hours.  ↑Reduction in some self-report symptoms |  | |
|  |  |  | 60.1 ±1.6 | 65 ±2.0 |  |  |  |  |  |  |  |  |  | |
|  |  |  |  |  |  |  |  |  |  |  |  |  |  |  |
| Fong et al.  (2014) (69) | Hong Kong | 23 | 11 | 12 |  |  | **Qigong** - 18 forms of Tai Chi Internal Qigong x 18 forms x 6mins. 1 session. | Slow | > 60 months post-surgery (Mean) | Circumference |  | ↔ Despite reductions in girth of the affected arm, there were no significant between the 2 groups or girth variables pre or post-test. |  | |
|  |  |  | 58.3 ±10.1 | 53.8 ±4.2 |  |  |  |  |  |  |  |  |  | |
|  |  |  | 20.8 ± 3.0 | 22.6 ± 3.3 |  |  |  |  |  |  |  |  |  |  |
| Buchan et al.  (2016) (57) | Australia | 41 | 20 | 21 |  |  | **RE of upper and lower body**. Weeks 1-4 2 x sets x 10-12reps. 150min total per week. Weeks 5-12: 2 sets x 8-10 reps.  150min total per week. Warm-up/cool down x 5mins each.  12 weeks  OR **AE (participant choice**, e.g. walking, jogging, cycling swimming). | Light progressed to moderate. | > 48 months post diagnosis (Mean) | Circumference.  BIS.  Self-report (Norman questionnaire) | QOL.  Strength. Endurance. Function/  disabilities.  6 min walk test. Qualitative.  PA levels at baseline as per guidelines. | ↑Trend for lymphoedema symptom severity to decline in both groups by week 12. ↓ Increased swelling, heaviness and/or aching was reported by 72% during the intervention but this was not attributed to the interventions. | ↑RE group significant improvement in upper body strength. ↑Both groups saw improvements in muscular endurance, aerobic fitness and QOL. | |
|  |  | Mean (95% CI) | | | | |  |  |  |  |  |  | Total PA at baseline: 48.8% sufficient. 39% insufficient and 12.2% sedentary. | |
|  |  | 56.0 (52.8-59.2) | 58.5 (54.2-62.8) | 53.7 (48.9-58.5) |  |  |  |  |  |  |  |  | ↑*Participants reported that lymphoedema was stable during the exercise program, despite ordinarily being unstable (worse) during the summer* | |
|  |  | 29 (27–32) | 30 (26–33) | 29 (26–32) |  |  |  |  |  |  |  |  |  |  |
| Singh et al.  (2016) (110) | Australia | 41 | 15 | 24 |  |  | **RE of upper and lower body.** Weeks 1-4 2 x sets x 10-12reps. 150min total per week. Weeks 5-12: 2 sets x 8-10 reps.150min total per week. Warm-up/cool down x 5mins each.  12 weeks  **OR AE (participant choice,** e.g. walking, jogging, cycling swimming). | Light progressed to moderate. | > 60 months post diagnosis (Mean) | Circumference. BIS. Self-report (rat  ed mild, moderate, severe) | PA levels collected via survey but not reported on. Subjective reasons for not wearing compression. | ↑Reduction in symptom severity irrespective of garment use | *Various reasons reported for not wearing compression garment including dislike. Reasons for wearing it included normal practice (n=5), it helped to reduce fluid during exercise (n=3), prevented the feeling of increased swelling (n=1)* | |
|  |  |  | 52.7 ±9.4 | 59.1 ±9.8 |  |  |  |  |  |  |  |  |  |  |
|  |  |  | 29.2 ±5.5 | 30.3 ±7.1 |  |  |  |  |  |  |  |  |  |  |
| Lacorossi et al.  (2019)(85) | Italy | 100 | 50 | 50 |  |  | **Dragon boat racing**. 2 x per week x 6months |  |  | Circumference. Self-report (BR23) | QOL. | ↑Marked improvement in lymphoedema symptoms after exercise in the dragon boat racing group. | ↑Higher QOL, reduced in fatigue, insomnia, dyspnoea in dragon boat group.  ↑Significant reduction in physical related disorders, emotional, cognitive, and relational/role in dragon boat group. | |
|  |  |  | < 40: 0% 41-50: 22% 51-60: 32% >60:46% | < 40: 2% 41-50: 36% 51-60: 48% >60:14% |  |  |  |  |  |  |  |  |  |  |
|  |  |  | 25.83 ±5.16 | 22.56 ±2.71 |  |  |  |  |  |  |  |  |  |  |
| Atef et al.  (2020) (48) | Egypt | 30 | 15 | 15 |  |  | **Virtual reality** -tennis, triceps extension, rhythmic boxing. 30mins with Nintendo Wii.  1 min break between games.  2 x per week x 4 weeks. |  | > 6 months post-surgery | Circumference | Function/  disabilities. | ↑ Both PNF and VR, showed significant differences in limb volume after 4 weeks.  ↔ Age, BMI and number of LN removed showed no significant effect on limb volume | ↑ Both groups showed significant differences in arm function after 4 weeks. | |
|  |  |  | 54.07 ±8.28 | 53.07 ±7.24 |  |  |  |  |  |  |  |  |  |  |
|  |  |  | 34.56 ± 7.65 | 35.73 ± 4.71 |  |  |  |  |  |  |  |  |  |  |
| Boeer et al.  (2023) (54) | Germany | 98 | 28 | 70 |  |  | **Dragon boat paddling** x 1.5hrs x 1 x per week x 6months |  | > 48 months post-surgery (Mean) | Circumference | QOL.  Pain. | ↔Lymphoedema incidence was not significant between groups. | ↑The global health scale among paddlers significantly improved from the beginning of the study to the end of the study | |
|  |  |  | 60.0 ±9.2 | 62.5 ±10.2 |  |  |  |  |  |  |  |  |  |  |
|  |  |  | 24.2 ±3.1 | 26.3 ±4.7 |  |  |  |  |  |  |  |  |  |  |
| Damato et al.  (2023) (62) | Russia | 35 | 18 | 17 |  |  | **RE of upper and lower body** x 20-25mins.  4 x per week x 6 weeks. | Vigorous to very hard. |  | Circumference | QOL. Strength. Function/  disabilities.  Level of PA scale. | ↑Significant decrease in arm circumference at 4 out of 5 points in IG at 6 weeks. | ↑Significant difference between groups for strength.  ↑Significant reduction for QOL and disabilities score.  ↔ Level of PA scale measured at baseline. No participants sedentary. | |
|  |  |  | 52.2 ±10.9 | 56.6 ±10.4 |  |  |  |  |  |  |  |  |  |  |
|  |  |  | 25.5 ±3.6 | 25.9 ±3.4 |  |  |  |  |  |  |  |  |  |  |
| Cohort (n=2) | | | | | | | | | | | | | |  |
| Douglass et al.  (2012) (66) | Australia | 18 | 9 | 9 |  |  | **Yog**a (with heavy loading the affected side). Daily - 90mins x 1 day and 40mins x 6 days. 4 weeks. Follow up at 6months. |  |  | BIS.  Perometry.  Tonometry.  Self-report (NRS) | QOL. | ↔No statistically significant differences between groups at 6months. | ↔QOL score declined in both groups but less in the group that continued Yoga. | |
|  |  |  | 65.0 ±12.4 | 60.4 ±11.1 |  |  |  |  |  |  |  |  |  |  |
|  |  |  | 30.2 ±4.6 | 31.5 ±4.5 |  |  |  |  |  |  |  |  |  |  |
| Bloomquist et al.  (2014) (51) | Denmark | 149 |  |  |  |  | **High intensity AE** (static bikes) and **RE of upper and lower body** with machines plus aerobic warm-up. 2hours x 3 x per week x 6 weeks. This is the 4-26month follow up. | AE: Vigorous RE: Progressive Vigorous - Near Max | < 6 months post-surgery (Median) | Circumference.  Self-report (if diagnosed with BCRL or not) | Strength.  Leisure time PA questionnaire. | ↔ No association between performing heavy RE during adjuvant therapy and the development of BCRL.  ↓More participants in the group diagnosed with BCRL were overweight and had radiotherapy and ALND. | ↑ Self-reported PA levels increased at 4-26month follow up in those with and without BCRL. | |
|  |  | 47.7 |  |  |  |  |  |  |  |  |  |  |  |  |
|  |  | 24.1 |  |  |  |  |  |  |  |  |  |  |  |  |
| Longitudinal follow-up of a randomised controlled trial (n=1) | | | | | | | | | | | | | |  |
| Tidhar et al.  (2004) (113) | Israel | 3 | 1 | 1 | 1 |  | **Aquatic therapy** x 60mins x 1-2x per week x 14months. | Slow pace | > 48 months post diagnosis | Circumference | Feedback comments. | ↑No lymphoedema exacerbations and all demonstrated further affected limb volume reductions. | *↑Arm stronger and more confident using it.* | |
|  |  |  | 57 | 55 | 63 |  |  |  |  |  |  |  |  |  |
|  |  |  |  |  |  |  |  |  |  |  |  |  |  |  |
| Case-report (n=1) | | | | | | | | | | | | | |  |
| Sagen et al.  (2009) (102) | Norway | 204 | 104 | 100 |  |  | **RE of upper body** x 15reps of each exercise x 45mins.  2-3 x per week x 6months x 2 years. | Low and progressed to no upper limit | Recruited at time of surgery | Water displacement. Self-report (VAS). | Pain. Upper limb PA questionnaire. | ↔Arm volume significantly increased after 2 years but did not differ between groups.  ↓Individuals with a baseline BMI >25 had a significantly increased risk of BCRL at 2years | ↓ Pain and heaviness were significantly higher in the affected limb for the no restriction in PA group compared to the restricted PA group.  ↔The group with no restricted PA had significantly higher home PA levels at 3 and 6 months but the PA levels did not differ between groups at 2years. | |
|  |  |  | 54 ±90.6 | 55 ±90.5 |  |  |  |  |  |  |  |  |  |  |
|  |  | Recorded for analysis but not reported on as baseline data. | | | | |  |  |  |  |  |  |  |  |
