# Supplementary material for: Core components of physical activity interventions in the prevention and management of breast cancer-related lymphoedema (BCRL): A scoping review
Source: PLoS One. 2026 Sep 2;21(9):e0355189. doi: 10.1371/journal.pone.0355189 (PMC13537585; doi:10.1371/journal.pone.0355189)
Supplement: Supplementary file 6 [file pone.0355189.s009.docx]

First, I would like to thank you for the opportunity to review this manuscript, which addresses an interesting topic that aligns closely with emerging research in our laboratory, namely lymphedema. Overall, I find the subject timely and relevant. However, in its current form, the manuscript reads more like a thesis than a journal article.

While comprehensive reviews are necessarily detailed, this manuscript would benefit from substantial simplification and condensation. In particular, two main aspects should be addressed:

1) Reduction and streamlining of the text. The current level of detail is excessive, which negatively affects readability and, at times, makes the manuscript difficult to follow. A more concise presentation would greatly improve clarity and reader engagement.
2) Improved organization of figures and tables. I recommend limiting the main manuscript to one central figure (e.g., a PRISMA flow diagram) and two key tables (search terms and study summary). Additional figures and tables could be moved to supplementary materials, ideally restricted to no more than two or three supplementary tables and two to three supplementary figures.

In summary, the manuscript presents a solid and valuable story; however, it would benefit from being conveyed in a more focused, organized, and streamlined manner. As it stands, the abundance of detail hinders the development of a clear and fluid narrative. I would welcome the opportunity to review a revised version, at which point more specific and detailed suggestions could be provided to further strengthen the manuscript.

**Introduction + Objectives**

**Comment 1**: Overall, while the introduction is informative, it is excessively long. I recommend that the authors synthesize the content and focus more directly on the problem under investigation. A clearer and more effective structure for the introduction could be as follows (3-4 paragraphs max):

1) Provide a brief contextualization of breast cancer and lymphedema, including key epidemiological data and main symptoms, without an overly detailed description of aspects such as disease staging.
2) Emphasize the role of exercise and physical activity in individuals with lymphedema. Important points already mentioned, such as the historical myth regarding the risks of exercise and the current body of evidence supporting its safety and benefits, should be retained, but presented more succinctly, focusing on an aggregated synthesis of study findings. Defining physical activity is unnecessary, and this paragraph should be removed.
3) Clearly identify the gaps in the literature and, based on these, formulate the study objectives. It is not necessary to explain what a scoping review is; instead, the objectives should be stated in a clear, concise, and focused manner.

**Comment 2:** After reviewing the article cited to support the percentage of breast cancer-related lymphedema incidence, it seems that the reported value does not specifically represent the proportion of patients with this condition. Instead, it appears to refer to patients who underwent ALND and RLNR and subsequently developed lymphedema. Clarification of the patient population to which the reported percentage applies would help avoid misinterpretation.

**Comment 3:** The sentence in line 89 may not be optimally phrased in its current form, particularly when considered in the context of the preceding sentence.

**Methods**

**Comment 4**: What type of study quality assessment was performed? A quality assessment must be added and conducted according to the designs of the included studies.

- ***Inclusion Criteria***

**Comment 5**: At its first mention, PCC should be clearly defined as Population, Concept, and Context.

- ***Concept***

**Comment 6**: Please clarify the type of physical activity included. Based on the terminology used, it is unclear whether only general physical activity (e.g., walking) was considered or whether structured aerobic and strength-training exercise interventions were also included. Based on the abstract, it appears that the review included not only physical activity but also exercise interventions. Were yoga or dance interventions included?

- ***Context***

**Comment 7**: Remove this part. No important information here.

- ***Limitations***

**Comment 8**: How many papers were excluded based on this criterion?

**Results**

**Comment 9**: Figure 1 – revise calculations. Numbers seem to be incorrect.

**Comment 10**: Line 294 – 91.? It seems that a number is missing

**Comment 11:** From the perspective of both a reader and a reviewer, the Results section is currently overly long and difficult to follow. While the section can be initiated, it is challenging to maintain clarity throughout, and by the midpoint of reading it becomes difficult to retain the key findings.

To improve readability and synthesis, I strongly recommend including a comprehensive table that aggregates the most relevant information needed to understand the included studies and their outcomes. A table with approximately 6–7 columns, organized by study design (as currently done in Table S1), would be particularly helpful. Suggested columns include:

1. Author, year, and country
2. Sample size (overall and per group), including mean age and standard deviation
3. Timing of the intervention (e.g., at diagnosis, during adjuvant treatment, post-treatment)
4. Intervention characteristics, clearly describing physical activity/exercise parameters (frequency, intensity, time/duration, and type)
5. Method used to assess or measure lymphedema
6. Outcomes of interest
7. Main results observed (including within- and/or between-group differences, when applicable)

In essence, this table could represent an expanded and improved version of the current Table 1, incorporating the information presented in Table 2 and including all studies. This table would serve as the primary resource guiding readers toward the most critical aspects of the evidence. The accompanying text should then focus on directing readers to the relevant tables and figures that summarize key findings, rather than describing every individual result in detail, which currently makes the section difficult to follow.

Additional information such as funding sources, researcher characteristics, guidelines used etc etc etc, while valuable, could be consolidated into a single separate table, provided as supplementary material, and more important removed from the text.
