# Supplementary material for: Core components of physical activity interventions in the prevention and management of breast cancer-related lymphoedema (BCRL): A scoping review
Source: PLoS One. 2026 Sep 2;21(9):e0355189. doi: 10.1371/journal.pone.0355189 (PMC13537585; doi:10.1371/journal.pone.0355189)
Supplement: Supplementary file 7 [file pone.0355189.s011.docx]

06-03-2026

Dear Academic Editor and Reviewer,

Thank you for your very welcomed and thorough comments to our recent submission to PLOS One. The manuscript has been amended accordingly. All changes are summarised in the table below:

| **Academic Editor Comments** | |
| --- | --- |
| **Comment number, summary** | **Amendment** |
| Comment 1:  Please ensure that your manuscript meets PLOS ONE's style requirements, including those for file naming. | - Headings have been amended to 18 font bold for Level 1, 16 font bold for Level 2 and 14 font bold for Level 3. - Files included have been named as advised - Tables inserted are cell based in Microsoft word. - Title presentation has been revised. |
| Comment 2:  Competing Interests section: Please confirm that this does not alter your adherence to all PLOS ONE policies on sharing data and materials, by including the following statement. | This has been confirmed on the cover letter to be updated on the competing interests section of the submitted form. |
| Comment 3:  We note that Figure 2 in your submission contain [map/satellite] images which may be copyrighted. | This figure has been removed. |
| Comment 4:  If the reviewer comments include a recommendation to cite specific previously published works, please review and evaluate these publications to determine whether they are relevant and should be cited. There is no requirement to cite these works unless the editor has indicated otherwise. | The reviewer has not recommended to cite specific previously published works. |
|  | |
| **Reviewer Comments** | |
| **Comment number, summary** | **Amendment** |
| **Introduction and Objectives** | |
| Comment 1:  Overall, while the introduction is informative, it is excessively long. I recommend that the authors synthesize the content and focus more directly on the problem under investigation. | The authors would like to thank the reviewer for this suggested improvement.   - The introduction has been condensed to 3 paragraphs, concluding with one aim and five objectives clearly defined. - Word count has been reduced. - Unnecessary points and definitions as suggested have been removed. |
| Comment 2:  After reviewing the article cited to support the percentage of breast cancer-related lymphedema incidence, it seems that the reported value does not specifically represent the proportion of patients with this condition. Instead, it appears to refer to patients who underwent ALND and RLNR and subsequently developed lymphedema. Clarification of the patient population to which the reported percentage applies would help avoid misinterpretation. | The authors would like to thank the reviewer for this suggested improvement.  Breast cancer-related lymphoedema incidence wording has been amended to account for the five-year cumulative rate incidence for those that receive both axillary lymph node dissection (ALND) and regional lymph node radiation (RLNR):  ‘Research reports that up to 30.1% of breast cancer survivors (BCS) who receive both ALND and RLNR, will experience BCRL in the five years post treatment.’ |
| Comment 3:  The sentence in line 89 may not be optimally phrased in its current form, particularly when considered in the context of the preceding sentence. | Following the restructuring of the introduction to make it more concise, the sentence ‘avoidance and overprotection behaviours should now be discouraged’ in line 89 has been removed. |
| **Methods** | |
| Comment 4:  What type of study quality assessment was performed? A quality assessment must be added and conducted according to the designs of the included studies. | The authors would like to thank the reviewer for this suggested clarification.  A study quality assessment was not performed as in accordance with Joanna Briggs Institute (JBI) methodology which was used to guide this scoping review. As noted in their document cited below ‘an assessment of methodological limitations or risk of bias of the evidence included within a scoping review is generally not performed’. (JBI, 2024)  <https://jbi-global.atlassian.net/wiki/spaces/MANUAL/pages/355862553/10.1.1+Why+a+scoping+review> |
| Comment 5:  At its first mention, PCC should be clearly defined as Population, Concept, and Context. | PCC has been defined as Population, Concept, Context at its first mention. |
| Comment 6:  Please clarify the type of physical activity included. | The authors would like to thank the reviewer for this suggested clarification.  The type of physical activity has been clarified as both structured and unstructured:  ‘Concept: Studies that included all types of PA, both structured and unstructured, as the main primary intervention and stated a recognised outcome measure for lymphoedema.’ |
| Comment 7:  Remove this part. No important information here. | In keeping with Participant, Concept, Context (PCC) questions development framework used to inform the eligibility criteria as per Joanna Briggs Institute guidance, including ‘Context’ was a required. Therefore it has not been removed, but edited to provide greater clarification:  Context: Any environment or setting (e.g. clinical, home community, online). |
| Comment 8: How many papers were excluded based on this criterion? | The subheadings of ‘limitations’ and ‘types of sources’ have been removed from the inclusion criteria section and incorporated into the search section as when performing the scoping review they were part of the search strategy.  Limiting the search strategy to English language articles has been recognised as an overall study limitation at the end of the manuscript. |
| **Results** | |
| Comment 9:  Figure 1 – revise calculations. Numbers seem to be incorrect. | Thank you for drawing our attention to this error. All PRISMA calculations have been revised and new PRISMA document (Fig 1) is presented in accordance with screening software used, Covidence. |
| Comment 10:  Line 294 – 91.? It seems that a number is missing | With the restructuring of the results section this figure has been removed. |
| Comment 11:  From the perspective of both a reader and a reviewer, the Results section is currently overly long and difficult to follow. While the section can be initiated, it is challenging to maintain clarity throughout, and by the midpoint of reading it becomes difficult to retain the key findings. | Thank you for your expert feedback which has enhanced this section. The results and discussion sections have been edited to reduce the volume of information and improve conciseness.  Main amendments are:   - Table 2 has been presented with the most relevant information, using the headings as you have suggested and also including BMI (overall and group). - Additional information has been removed from the text to supplementary tables and figures. - Paragraphs have been reordered and reported to directly align to each of the objectives. For example:  i. Participant characteristics: This section has been simplified to identify key risk factors only i.e. axillary node dissection, radiotherapy, BMI, chemotherapy. ii. PA intervention characteristics: Excessive information has been removed, focus has been centred around the frequency, intensity, time and type of the interventions and the CERT reporting template. iii. Outcome measures: This section has been briefly edited. iv. Participants’ PA behaviours: Attention has been paid to PA behaviours at the start, during and end of intervention, as defined in the initial aim. Excessive detail around adherence rates etc has been removed.   The final objective to identify knowledge gaps and areas for further research has been addressed throughout the discussion section. The discussion section has been edited to reflect only the key findings. |
