# Supplementary material for: Core components of physical activity interventions in the prevention and management of breast cancer-related lymphoedema (BCRL): A scoping review
Source: PLoS One. 2026 Sep 2;21(9):e0355189. doi: 10.1371/journal.pone.0355189 (PMC13537585; doi:10.1371/journal.pone.0355189)
Supplement: Supplementary file 8 [file pone.0355189.s012.docx]

**Response to Reviewers**

Dear Academic Editor and Reviewers 2, 3 and 4.

Thank you for your very welcomed and thorough comments to our recent resubmission to PLOS One. The manuscript has been amended accordingly. All changes are summarised in the table below. Note underlined text has been taken directly from the manuscript.

**Abstract**

| **Academic Editor** | **Reviewer 2** | **Reviewer 3** | **Reviewer 4** |
| --- | --- | --- | --- |
| **Comment 1:**  "Motivational strategies (34.2%) or behavioural change theory (6.3%) to encourage physical activity participation were infrequently included". For the motivational strategies, I don't think 34.2% is regarded as infrequently included. Suggest not to interpreting it as "infrequent", but only report the figure. |  | **Comment 1:**  I would suggest regarding the results section, you present the number of studies together with the percentages. |  |
| **Author response:**  The authors would like to thank the academic editor for this recommendation. We have removed the word ‘infrequent’ from 34.2%. |  | **Author response:**  The authors would like to thank the reviewer for this comment. We have included the number of studies together with the percentages. |  |
| **Comment 2:**   "five future research recommendations have been proposed". It is suggested illustrating these recommendations briefly to address the last objective of the review if word limit doesn't exceed. |  | **Comment 2:**  Provide at summary of least one key recommendation in the conclusion section. |  |
| **Author response:**  The authors thank the academic editor for this suggestion and recognise the importance of illustrating the recommendations within the abstract. Therefore these have been briefly summarised in conclusion section of the abstract. However, in order to keep within the 300 word limit (current word limit is 297), there have been other brief amendments to the abstract. |  | **Author response:**  The authors thank the reviewer for this suggestion and recognise the importance of including the recommendations within the abstract. Therefore these have been briefly summarised in conclusion section of the abstract. However, in order to keep within the 300 word limit (current word limit is 297), there have been other brief amendments to the abstract. |  |

**Introduction**

| **Academic Editor** | **Reviewer 2** | **Reviewer 3** | **Reviewer 4** |
| --- | --- | --- | --- |
|  | **Comment 1:**  There is confusion between the terms “physical activity” and “exercise,” which are used interchangeably throughout the manuscript. The authors should clearly define both terms and use them consistently. | **Comment 3:**  Check the transition (logical flow) from line 71 through to 74. | **Comment 1:**  Line 89 (hanging sentence): With the fear of exacerbating swelling often cited as a barrier to PA (14). |
|  | **Author response:**  The authors thank the reviewer for this comment. With both structured and unstructured physical activity interventions being included in this review we acknowledge that there is crossover in the terminology. In order to separate the terms as best as possible we have including the following:   - *Introduction (Rationale)*   We have included the definition: Physical activity (PA) commonly defined as “any bodily movement…..requires energy expenditure” (5)….   - *Methods (data charting)*   We have defined exercise as planned, structured and repetitive, and a subset of PA. This provides the rationale for including the CERT template to provide further clarity. This is written in the manuscript as:  From the initial review of the included studies, it was identified that most of the included interventions were planned, structured and repetitive, thus resembling exercise which is defined as a subset of PA (36).   - *Strengths and limitations*   The inclusion of structured and unstructured PA has been recognised as a strength of the study. This is written in the manuscript as:  Incorporating both structured and unstructured PA interventions into this scoping review enabled a comprehensive overview of both PA and exercise research in this area. | **Author response:**  The authors thank you for this comment and we have addressed the logical flow in the manuscript as follows:  BCRL typically presents as swelling of the upper limb, chest wall or trunk and may be associated with heaviness, stiffness, numbness, tightness, fatigue, weakness and decreased coordination and mobility in the affected arm (3). | **Author response:**  The authors thank you for your comment. We have removed that line from the manuscript. |
|  |  |  | **Comment 2:**  Line 102 and 103 (poor punctuation): PA is commonly defined as ‘any bodily movement produced by skeletal muscles that results in energy expenditure’ (22), however, the term alone has been challenged for… |
|  |  |  | **Author response:**  The authors thank you for this comment. We have amended the PA definition to:  Physical activity (PA) commonly defined as “any bodily movement….. that requires energy expenditure”. |

**Methods**

| **Academic Editor** | **Reviewer 2** | **Reviewer 3** | **Reviewer 4** |
| --- | --- | --- | --- |
|  | **Comment 2:**  The justification for choosing a scoping review needs to be clearer, explaining why this approach is more suitable than a systematic review. | **Comment 3:**  With respect to “Joanna Brigg’s Institute (JBI) methodology” be specific “Joanna Brigg’s Institute (JBI) methodology for conducting scoping reviews” |  |
|  | **Author response:**  The authors thank you for this comment and do feel that providing justification for choosing a scoping review over a systematic review strengthens the methodology section of the manuscript. We have therefore added in:  A scoping review which aims to map multiple types of evidence, was felt most appropriate in order to identify gaps and priorities for further research (32). | **Author response:**  The authors thank you for bringing our attention to this detail to strengthen the methodology section. We have therefore added in your suggestion as follows:  A systematic scoping review conducted in accordance with the Joanna Brigg’s Institute (JBI) methodology for conducting scoping reviews (31) was chosen to explore and synthesise the literature regarding PA and BCRL. |  |
|  | **Comment 3:**  The search strategy also requires better transparency by providing full details for all databases and clarifying how grey literature was searched. | **Comment 4:**  Search I would suggest making it “Search Strategy” |  |
|  | **Author response:**  The authors thank you for this suggestion in regards to transparency and clarity of the search strategy. The search strategy for Medline is presented in Table 1 of the main manuscript and it is stated that the primary database searches across CINAHL and Medline were translated across the other three databases Embase, SportsDiscus and Scopus.  In response to your welcomed feedback and in order to keep the manuscript concise for the reader, we have placed further data in the supplementary section. This has data includes:  - The search strategy for the other primary search database, CINAHL, as a supplementary table (S5_Table).  - Table detailing the websites of the key societies searched in the grey literature search (S4_Table). | **Author response:**  Thank you again for bringing the authors attention to this detail and we have amended search to search strategy as suggested. |  |
|  | **Comment 4:**  Please send the PRISMA checklist file at your earliest convenience. In addition, include a clear statement in the Methods section confirming compliance with PRISMA guidelines and provide registration details (if applicable). | **Comment 5:**  Study Selection What do you mean by “randomly selected articles?” |  |
|  | **Author response:**  The authors thank you for your query in regards to addressing the methodological reporting requirements.  We have stated in the main manuscript Methods section that:  This approach was reported in line with the PRISMA-ScR (Preferred Reporting Items for Systematic reviews and Meta-Analysis extension for Scoping Reviews) checklist (S1 Table) (33).  We have submitted S1 Table as supplementary data in our submission to PLOS ONE.  The study has not been registered with PRISMA but with Open Science Framework. We have included a statement in regard to this under the Protocol and Registration section:  The scoping review protocol was registered with Open Science Framework on 25-11-24, updated on 22-01-25 and can be found at: <https://doi.org/10.17605/OSF.IO/T6CUQ>. | **Author response:**  The authors would like to thank you for bringing our attention to the use of the word randomly and appreciate that it is not self-explanatory. To provide clarity to the reader of this scoping review we have amended this statement:  Two reviewers (LH and JSW) independently pilot screened the title and abstract of the first 25 articles, ordered by their Covidence ID i.e. the ID assigned to them via the software Covidence. |  |
|  |  | **Comment 6:**  Study Selection  • Did you include the three papers that had missing data or further data was required? If yes, it would be important to provide a rationale why these studies were included in the review and whether the missing data had an impact on the overall outcome of the review. |  |
|  |  | **Author response:**  The authors thank you for your expert feedback we have added this detail to the data charting section to include:  Where data was missing or when additional data was required, the authors of five papers were contacted. Two authors responded and clearly answered the questions (40, 41), two authors were not contactable but the data from their studies was included in the scoping review as the authors agreed that comprehensive data charting could still take place (42, 43). The final author contacted did not respond to email contact and as data from this study could not be comprehensively charted, it was excluded from the final number of studies for review.  In addition we have acknowledged the missing data from one study in the limitations section. |  |

**Results**

| **Academic Editor** | **Reviewer 2** | **Reviewer 3** | **Reviewer 4** |
| --- | --- | --- | --- |
| **Comment 3:**  "the total number of studies (n=79) will be used ...." Past tense should be used instead of future tense. | **Comment 5:**  The results are mainly descriptive, relying on percentages and counts, with limited deeper analysis. The paper would benefit from more interpretation and grouping of findings to provide meaningful insights. | **Comment 7:**  Table 2 For the randomized controlled trial studies, it would be important to communicate to readers which group served as the control or intervention. |  |
| **Author response:**  The authors thank you for this comment and have amended the tense accordingly:  Unless noted, the total number of studies (n=79) was used as the denominator to calculate percentages. | **Author response:**  The authors thank you for your comment and feel by addressing it this has strengthened the results section. In keeping with the JBI methodology for scoping reviews guidance we have kept the original headings to ensure the results align specifically with the objective and scope of the review. An example of changes include:  Participants received a range of breast cancer treatments (S2 Fig.). Considering the risk factors associated with BCRL, it is not surprising that the majority of studies reported participants received surgery to the axillary nodes (n=64; 81.0%) (36,38-55,57,60,62,66,68-71,73,74,77-87,89-92,94-102,104-114), radiotherapy (n=55; 69.6%) (37-41,45,46,48-56,61,64,66,68-72,74-82,84,85,87,89,91,92,94,96,98-102,105-112,114)and chemotherapy (n=54; 68.4%) (37-41,44-48,50-56,59,64,66,68-72,77-80,82-85,87,89-92,94,96-98,100-102,105-112,114). | **Author response:**  Thank you for this comment which provides clarity to the reader. We have now added into the notes section below Table 2 the following:  For the randomised controlled trials (RCT) group A is the intervention group and Group B is the control group except when there is more than one intervention group. In this case if three groups are reported Group A, Group B are the intervention groups and Group C is the control group. If four groups are reported then Group A, Group B, Group C are the intervention groups and Group D is the control group. |  |
| **Comment 4:**  Some formatting issues should be corrected, including space between 50-60 years (line 368, page 39), 60 months (5 years) (line 664, page 56), twelve should be presented as 12 (line 689, page 57). |  | **Comment 8:**  Table 2  The authors, you indicated just a name for all the studies. Does that mean each study was conducted by just one researcher? If that is not the case, I would suggest you revise it |  |
| **Author response:**  Thank you for your feedback. We have addressed these formatting issues within the manuscript. |  | **Author response:**  Thank you for bringing our attention to this omission. The authors welcome your feedback and have amended this accordingly. |  |
| **Comment 5:**   In line 725, page 58, "The use of CERT identified that only 34.2% ...." Suggest deleting the word "only". |  |  |  |
| **Author response:**  Thank you for comment the authors have now removed the word only. |  |  |  |

**Discussion and Areas for Further Research**

| **Academic Editor** | **Reviewer 2** | **Reviewer 3** | **Reviewer 4** |
| --- | --- | --- | --- |
|  | **Comment 6:**  The discussion on behavioural change theory is also underdeveloped, despite being an important finding, and should be expanded. Similarly, outcome measures for lymphoedema vary widely across studies, but this is not fully analyzed or compared in a structured way. | **Comment 9:**  The statement “clinical practice guidelines now recommend that PA is safe for this population” (line 337-338) needs to be referenced. |  |
|  | **Author response:**  The authors thank you for your comments regarding the behavioural change theory and lymphoedema outcomes. We have now restructured both these within the manuscript. For example:  With most interventions instead based primarily upon scientific (63.8%) and/or physiological rationale (43%) it is important to recognise that PA behaviour has been negatively associated with a BCRL diagnosis. For example, in a 5 year longitudinal study, PA levels (self-reported as regular, occasional or none) of BCS were found to be significantly (p=0.004) lower in those with a BCRL diagnosis when compared to those without a BCRL diagnosis (126). These findings, alongside the infrequent reporting of intervention acceptability (17.7%) and the inconsistent reporting of PA levels at baseline or throughout the studies, highlights a limited understanding of PA behaviours….. | **Author response:**  The authors thank you for bringing our attention to this detail and we have referenced this statement appropriately. |  |
|  | **Comment 7:**  The discussion section…would benefit from clearer structuring. The authors are encouraged to more explicitly highlight key convergences, divergences, remaining gaps in the literature, and implications for clinical practice. This section is quite lengthy and could be improved by making it more concise and focused. | **Comment 10:**  Line 340, were all the studies included interventional studies? |  |
|  | **Author response:**  Thank you for this feedback regarding the discussion section. The authors have reviewed this section and restructured it. For example:  The participants most frequently included in the interventions were 50 - 60 years old females (58.2%), diagnosed with stage II breast cancer (21.5%), had received the breast cancer treatments axillary node surgery (81%), radiotherapy (69.6%) and chemotherapy (68.4%), and were overweight or obese (65.8%). These demographics represent those most at risk of BCRL (122,123) and whilst this may reflect those easiest to recruit to such studies, it does highlight gaps within the literature regarding understudied populations. For example, around 390 men are diagnosed with breast cancer each year in the UK and this population was not adequately represented (122). | **Author response:**  Thank you for your comment in regards to the use of the word intervention. In the inclusion criteria physical activity had to be the main primary intervention of the study so therefore all included studies were PA interventions. We have reworded this line as follows to provide more clarity:  However, in order to further understand how research in this area could be translated into real-life scenarios, this scoping review explored the core components of 79 PA intervention studies for those at risk of, or living with, BCRL from across the globe. |  |
|  |  | **Comment 11:**  I would suggest you refine Line 358 |  |
|  |  | **Author response:**  Thank you for your comment in regards to this line. The authors have restructured this paragraph, including this line in response to other peer reviewer comments. |  |
|  |  | **Comment 12:**  Why do you think the time for inculcating physical activity in the treatment of breast cancer varied? What has previous literature found? |  |
|  |  | **Author response:**  The authors thank you for your question and by addressing it add clarity to this section. We have now added into the manuscript the following:  Most studies recruited participants less than 6 months post diagnosis or surgery (26.6%) or beyond 60 months (5 years) post active treatment, surgery or diagnosis (26.6%). This may be attributed to studies investigating lymphoedema prevention recruiting participants early in the cancer timeline i.e. before BCRL develops and studies investigating lymphoedema management recruiting participants later in the cancer timeline i.e. when there is a confirmed diagnosis of BCRL. However BCRL risk is reported to peak between 12 and 30 months postoperatively (125). Therefore, future PA and BCRL research would benefit from longitudinal studies, recruiting participants from time of diagnosis and following them up for a number of years post treatment… |  |

**Strengths and Limitations**

| **Academic Editor** | **Reviewer 2** | **Reviewer 3** | **Reviewer 4** |
| --- | --- | --- | --- |
|  | **Comment 8:**  The limitations should be strengthened, particularly regarding language restriction (English only), lack of quality appraisal, and incomplete reporting of participant characteristics such as socioeconomic status and gender diversity. |  |  |
|  | **Author response:**  Thank you for your feedback in regards to the limitations section. We have addressed these points and included detail within the manuscript. In keeping with the JBI guidance for scoping reviews a quality appraisal was not required. |  |  |

**Conclusion**

| **Academic Editor** | **Reviewer 2** | **Reviewer 3** | **Reviewer 4** |
| --- | --- | --- | --- |
| **Comment 6:**  The fourth recommendations for future research "Identify effective psychological theories to enhance long-term PA participation in this population" (line 833, page 63). Why psychological theories should be identified, not behavioural change theories? Justification should be provided. | **Comment 9:**  ….could be more practical by emphasizing specific actions for researchers and clinicians. | **Comment 13:**  I would suggest having different headings for conclusions and recommendations |  |
| **Author response:**  Thank you for your comment. The authors have reviewed this statement and agree that behavioural change theories would be more appropriate and therefore have amended the text accordingly. | **Author response:**  Thank you for this comment. The authors have expanded the conclusion section and separated it from the recommendations which should provide more clarity in the actions for researchers and clinicians. | **Author response:**  The authors thank you for this comment and the conclusion and recommendations sections have been separated by different headings. |  |

**Reference List**

| **Academic Editor** | **Reviewer 2** | **Reviewer 3** | **Reviewer 4** |
| --- | --- | --- | --- |
|  | **Comment 10:**  …..the reference list is mostly relevant and up to date; however, a final check is recommended to ensure full compliance with the required referencing style and journal standards. Addressing these points will significantly improve the clarity, quality, and overall impact of the manuscript. |  |  |
|  | **Author response:**  Thank you for your comment. The authors have checked and amended the reference list. |  |  |
