# Supplementary material for: Core components of physical activity interventions in the prevention and management of breast cancer-related lymphoedema (BCRL): A scoping review
Source: PLoS One. 2026 Sep 2;21(9):e0355189. doi: 10.1371/journal.pone.0355189 (PMC13537585; doi:10.1371/journal.pone.0355189)
Supplement: Peer review history

# Peer review: Decision Letter 0

**DOI:** 10.1371/journal.pone.0355189.r001

**Authors:** Jen Edwards

22 Jan 2026

Dear Dr. Henry,

Thank you for submitting your manuscript to PLOS ONE. After careful consideration, we feel that it has merit but does not fully meet PLOS ONE’s publication criteria as it currently stands. Therefore, we invite you to submit a revised version of the manuscript that addresses the points raised during the review process.

Please submit your revised manuscript by Mar 08 2026 11:59PM. If you will need more time than this to complete your revisions, please reply to this message or contact the journal office at plosone@plos.org. When you're ready to submit your revision, log on to https://www.editorialmanager.com/pone/ and select the 'Submissions Needing Revision' folder to locate your manuscript file.

- A letter that responds to each point raised by the academic editor and reviewer(s). You should upload this letter as a separate file labeled 'Response to Reviewers'.
- A marked-up copy of your manuscript that highlights changes made to the original version. You should upload this as a separate file labeled 'Revised Manuscript with Track Changes'.
- An unmarked version of your revised paper without tracked changes. You should upload this as a separate file labeled 'Manuscript'.

If you would like to make changes to your financial disclosure, please include your updated statement in your cover letter. Guidelines for resubmitting your figure files are available below the reviewer comments at the end of this letter.

If applicable, we recommend that you deposit your laboratory protocols in protocols.io to enhance the reproducibility of your results. Protocols.io assigns your protocol its own identifier (DOI) so that it can be cited independently in the future. For instructions see: https://journals.plos.org/plosone/s/submission-guidelines#loc-laboratory-protocols. Additionally, PLOS ONE offers an option for publishing peer-reviewed Lab Protocol articles, which describe protocols hosted on protocols.io. Read more information on sharing protocols at https://plos.org/protocols?utm_medium=editorial-email&utm_source=authorletters&utm_campaign=protocols.

We look forward to receiving your revised manuscript.

Kind regards,

Jen Edwards

Staff Editor

PLOS One

Journal Requirements:

When submitting your revision, we need you to address these additional requirements.

1. Please ensure that your manuscript meets PLOS ONE's style requirements, including those for file naming. The PLOS ONE style templates can be found at

https://journals.plos.org/plosone/s/file?id=wjVg/PLOSOne_formatting_sample_main_body.pdf and

https://journals.plos.org/plosone/s/file?id=ba62/PLOSOne_formatting_sample_title_authors_affiliations.pdf

2. Thank you for stating the following in the Competing Interests section:

“LH- Received funding from The Department of the Economy Northern Ireland as part of wider PhD studies. However, the funders had no role in study design, data collection and analysis, decision to publish, or preparation of the manuscript.”

Please confirm that this does not alter your adherence to all PLOS ONE policies on sharing data and materials, by including the following statement: "This does not alter our adherence to PLOS ONE policies on sharing data and materials.” (as detailed online in our guide for authors http://journals.plos.org/plosone/s/competing-interests). If there are restrictions on sharing of data and/or materials, please state these. Please note that we cannot proceed with consideration of your article until this information has been declared.

Please include your updated Competing Interests statement in your cover letter; we will change the online submission form on your behalf.

3. We note that Figure 2 in your submission contain [map/satellite] images which may be copyrighted. All PLOS content is published under the Creative Commons Attribution License (CC BY 4.0), which means that the manuscript, images, and Supporting Information files will be freely available online, and any third party is permitted to access, download, copy, distribute, and use these materials in any way, even commercially, with proper attribution. For these reasons, we cannot publish previously copyrighted maps or satellite images created using proprietary data, such as Google software (Google Maps, Street View, and Earth). For more information, see our copyright guidelines: http://journals.plos.org/plosone/s/licenses-and-copyright.

We require you to either (1) present written permission from the copyright holder to publish these figures specifically under the CC BY 4.0 license, or (2) remove the figures from your submission:

1. You may seek permission from the original copyright holder of Figure 2 to publish the content specifically under the CC BY 4.0 license.

We recommend that you contact the original copyright holder with the Content Permission Form (http://journals.plos.org/plosone/s/file?id=7c09/content-permission-form.pdf) and the following text:

“I request permission for the open-access journal PLOS ONE to publish XXX under the Creative Commons Attribution License (CCAL) CC BY 4.0 (http://creativecommons.org/licenses/by/4.0/). Please be aware that this license allows unrestricted use and distribution, even commercially, by third parties. Please reply and provide explicit written permission to publish XXX under a CC BY license and complete the attached form.”

Please upload the completed Content Permission Form or other proof of granted permissions as an ""Other"" file with your submission.

In the figure caption of the copyrighted figure, please include the following text: “Reprinted from [ref] under a CC BY license, with permission from [name of publisher], original copyright [original copyright year].”

2. If you are unable to obtain permission from the original copyright holder to publish these figures under the CC BY 4.0 license or if the copyright holder’s requirements are incompatible with the CC BY 4.0 license, please either i) remove the figure or ii) supply a replacement figure that complies with the CC BY 4.0 license. Please check copyright information on all replacement figures and update the figure caption with source information. If applicable, please specify in the figure caption text when a figure is similar but not identical to the original image and is therefore for illustrative purposes only.

The following resources for replacing copyrighted map figures may be helpful:

USGS National Map Viewer (public domain): http://viewer.nationalmap.gov/viewer/

The Gateway to Astronaut Photography of Earth (public domain): http://eol.jsc.nasa.gov/sseop/clickmap/

Maps at the CIA (public domain): https://www.cia.gov/library/publications/the-world-factbook/index.html and https://www.cia.gov/library/publications/cia-maps-publications/index.html

NASA Earth Observatory (public domain): http://earthobservatory.nasa.gov/

Landsat: http://landsat.visibleearth.nasa.gov/

USGS EROS (Earth Resources Observatory and Science (EROS) Center) (public domain): http://eros.usgs.gov/#

Natural Earth (public domain): http://www.naturalearthdata.com/

4. If the reviewer comments include a recommendation to cite specific previously published works, please review and evaluate these publications to determine whether they are relevant and should be cited. There is no requirement to cite these works unless the editor has indicated otherwise.

[Note: HTML markup is below. Please do not edit.]

Reviewers' comments:

Reviewer's Responses to Questions

**Comments to the Author**

1. Is the manuscript technically sound, and do the data support the conclusions?

Reviewer #1: Partly

**********

2. Has the statistical analysis been performed appropriately and rigorously?

Reviewer #1: N/A

**********

3. Have the authors made all data underlying the findings in their manuscript fully available?

The PLOS Data policy

Reviewer #1: Yes

**********

4. Is the manuscript presented in an intelligible fashion and written in standard English?

Reviewer #1: No

**********

Reviewer #1: First, I would like to thank you for the opportunity to review this manuscript, which addresses an interesting topic that aligns closely with emerging research in our laboratory, namely lymphedema. Overall, I find the subject timely and relevant. However, in its current form, the manuscript reads more like a thesis than a journal article.

While comprehensive reviews are necessarily detailed, this manuscript would benefit from substantial simplification and condensation. In particular, two main aspects should be addressed:

1) Reduction and streamlining of the text. The current level of detail is excessive, which negatively affects readability and, at times, makes the manuscript difficult to follow. A more concise presentation would greatly improve clarity and reader engagement.

2) Improved organization of figures and tables. I recommend limiting the main manuscript to one central figure (e.g., a PRISMA flow diagram) and two key tables (search terms and study summary). Additional figures and tables could be moved to supplementary materials, ideally restricted to no more than two or three supplementary tables and two to three supplementary figures.

In summary, the manuscript presents a solid and valuable story; however, it would benefit from being conveyed in a more focused, organized, and streamlined manner. As it stands, the abundance of detail hinders the development of a clear and fluid narrative. I would welcome the opportunity to review a revised version, at which point more specific and detailed suggestions could be provided to further strengthen the manuscript.

Introduction + Objectives

Comment 1: Overall, while the introduction is informative, it is excessively long. I recommend that the authors synthesize the content and focus more directly on the problem under investigation. A clearer and more effective structure for the introduction could be as follows (3-4 paragraphs max):

1) Provide a brief contextualization of breast cancer and lymphedema, including key epidemiological data and main symptoms, without an overly detailed description of aspects such as disease staging.

2) Emphasize the role of exercise and physical activity in individuals with lymphedema. Important points already mentioned, such as the historical myth regarding the risks of exercise and the current body of evidence supporting its safety and benefits, should be retained, but presented more succinctly, focusing on an aggregated synthesis of study findings. Defining physical activity is unnecessary, and this paragraph should be removed.

3) Clearly identify the gaps in the literature and, based on these, formulate the study objectives. It is not necessary to explain what a scoping review is; instead, the objectives should be stated in a clear, concise, and focused manner.

Comment 2: After reviewing the article cited to support the percentage of breast cancer-related lymphedema incidence, it seems that the reported value does not specifically represent the proportion of patients with this condition. Instead, it appears to refer to patients who underwent ALND and RLNR and subsequently developed lymphedema. Clarification of the patient population to which the reported percentage applies would help avoid misinterpretation.

Comment 3: The sentence in line 89 may not be optimally phrased in its current form, particularly when considered in the context of the preceding sentence.

Methods

Comment 4: What type of study quality assessment was performed? A quality assessment must be added and conducted according to the designs of the included studies.

• Inclusion Criteria

Comment 5: At its first mention, PCC should be clearly defined as Population, Concept, and Context.

• Concept

Comment 6: Please clarify the type of physical activity included. Based on the terminology used, it is unclear whether only general physical activity (e.g., walking) was considered or whether structured aerobic and strength-training exercise interventions were also included. Based on the abstract, it appears that the review included not only physical activity but also exercise interventions. Were yoga or dance interventions included?

• Context

Comment 7: Remove this part. No important information here.

• Limitations

Comment 8: How many papers were excluded based on this criterion?

Results

Comment 9: Figure 1 – revise calculations. Numbers seem to be incorrect.

Comment 10: Line 294 – 91.? It seems that a number is missing

Comment 11: From the perspective of both a reader and a reviewer, the Results section is currently overly long and difficult to follow. While the section can be initiated, it is challenging to maintain clarity throughout, and by the midpoint of reading it becomes difficult to retain the key findings.

To improve readability and synthesis, I strongly recommend including a comprehensive table that aggregates the most relevant information needed to understand the included studies and their outcomes. A table with approximately 6–7 columns, organized by study design (as currently done in Table S1), would be particularly helpful. Suggested columns include:

1. Author, year, and country

2. Sample size (overall and per group), including mean age and standard deviation

3. Timing of the intervention (e.g., at diagnosis, during adjuvant treatment, post-treatment)

4. Intervention characteristics, clearly describing physical activity/exercise parameters (frequency, intensity, time/duration, and type)

5. Method used to assess or measure lymphedema

6. Outcomes of interest

7. Main results observed (including within- and/or between-group differences, when applicable)

In essence, this table could represent an expanded and improved version of the current Table 1, incorporating the information presented in Table 2 and including all studies. This table would serve as the primary resource guiding readers toward the most critical aspects of the evidence. The accompanying text should then focus on directing readers to the relevant tables and figures that summarize key findings, rather than describing every individual result in detail, which currently makes the section difficult to follow.

Additional information such as funding sources, researcher characteristics, guidelines used etc etc etc, while valuable, could be consolidated into a single separate table, provided as supplementary material, and more important removed from the text.

**********

what does this mean?). If published, this will include your full peer review and any attached files.

If you choose “no”, your identity will remain anonymous but your review may still be made public.

**Do you want your identity to be public for this peer review?**  For information about this choice, including consent withdrawal, please see our Privacy Policy

Reviewer #1: No

**********

[NOTE: If reviewer comments were submitted as an attachment file, they will be attached to this email and accessible via the submission site. Please log into your account, locate the manuscript record, and check for the action link "View Attachments". If this link does not appear, there are no attachment files.]

To ensure your figures meet our technical requirements, please review our figure guidelines: https://journals.plos.org/plosone/s/figures

You may also use PLOS’s free figure tool, NAAS, to help you prepare publication quality figures: https://journals.plos.org/plosone/s/figures#loc-tools-for-figure-preparation.

NAAS will assess whether your figures meet our technical requirements by comparing each figure against our figure specifications.

Attachment Submitted filename: Review.docx

# Author comment: Author response to Decision Letter 1

**DOI:** 10.1371/journal.pone.0355189.r002

6 Mar 2026

Academic Editor Comments:

Comment 1:

• Headings have been amended to 18 font bold for Level 1, 16 font bold for Level 2 and 14 font bold for Level 3.

• Files included have been named as advised

• Tables inserted are cell based in Microsoft word.

• Title presentation has been revised.

Comment 2

Statement included on Cover Letter, to be added to the Competing Interests Section of the submitted form, to confirm that this does not alter our adherence to all PLOS One policies on sharing data and materials.

Comment 3:

Figure has been removed

Comment 4:

The reviewer has not recommended to cite specific previously published works.

Reviewer Comments

Comment 1:

The authors would like to thank the reviewer for this suggested improvement.

• The introduction has been condensed to 3 paragraphs, concluding with one aim and five objectives clearly defined.

• Word count has been reduced.

• Unnecessary points and definitions as suggested have been removed.

Comment 2:

The authors would like to thank the reviewer for this suggested improvement.

Breast cancer-related lymphoedema incidence wording has been amended to account for the five-year cumulative rate incidence for those that receive both axillary lymph node dissection (ALND) and regional lymph node radiation (RLNR):

‘Research reports that up to 30.1% of breast cancer survivors (BCS) who receive both ALND and RLNR, will experience BCRL in the five years post treatment.’

Comment 3:

Following the restructuring of the introduction to make it more concise, the sentence ‘avoidance and overprotection behaviours should now be discouraged’ in line 89 has been removed.

Comment 4:

The authors would like to thank the reviewer for this suggested clarification.

A study quality assessment was not performed as in accordance with Joanna Briggs Institute (JBI) methodology which was used to guide this scoping review. As noted in their document cited below ‘an assessment of methodological limitations or risk of bias of the evidence included within a scoping review is generally not performed’. (JBI, 2024)

https://jbi-global.atlassian.net/wiki/spaces/MANUAL/pages/355862553/10.1.1+Why+a+scoping+review

Comment 5:

PCC has been defined as Population, Concept, Context at its first mention.

Comment 6:

The authors would like to thank the reviewer for this suggested clarification.

The type of physical activity has been clarified as both structured and unstructured:

‘Concept: Studies that included all types of PA, both structured and unstructured, as the main primary intervention and stated a recognised outcome measure for lymphoedema.’

Comment 7:

In keeping with Participant, Concept, Context (PCC) questions development framework used to inform the eligibility criteria as per Joanna Briggs Institute guidance, including ‘Context’ was a required. Therefore it has not been removed, but edited to provide greater clarification:

Context: Any environment or setting (e.g. clinical, home community, online).

Comment 8:

The subheadings of ‘limitations’ and ‘types of sources’ have been removed from the inclusion criteria section and incorporated into the search section as when performing the scoping review they were part of the search strategy.

Limiting the search strategy to English language articles has been recognised as an overall study limitation at the end of the manuscript.

Comment 9:

Thank you for drawing our attention to this error. All PRISMA calculations have been revised and new PRISMA document (Fig 1) is presented in accordance with screening software used, Covidence.

Comment 10:

With the restructuring of the results section this figure has been removed.

Comment 11:

Thank you for your expert feedback which has enhanced this section. The results and discussion sections have been edited to reduce the volume of information and improve conciseness.

Main amendments are:

• Table 2 has been presented with the most relevant information, using the headings as you have suggested and also including BMI (overall and group).

• Additional information has been removed from the text to supplementary tables and figures.

• Paragraphs have been reordered and reported to directly align to each of the objectives. For example:

i. Participant characteristics: This section has been simplified to identify key risk factors only i.e. axillary node dissection, radiotherapy, BMI, chemotherapy.

ii. PA intervention characteristics: Excessive information has been removed, focus has been centred around the frequency, intensity, time and type of the interventions and the CERT reporting template.

iii. Outcome measures: This section has been briefly edited.

iv. Participants’ PA behaviours: Attention has been paid to PA behaviours at the start, during and end of intervention, as defined in the initial aim. Excessive detail around adherence rates etc has been removed.

The final objective to identify knowledge gaps and areas for further research has been addressed throughout the discussion section. The discussion section has been edited to reflect only the key findings.

Attachment Submitted filename: Response to reviewers.docx

# Peer review: Decision Letter 1

**DOI:** 10.1371/journal.pone.0355189.r003

**Authors:** Ka Ming Chow

28 Apr 2026

Dear Dr. Henry,

Thank you for submitting your manuscript to PLOS ONE. After careful consideration, we feel that it has merit but does not fully meet PLOS ONE’s publication criteria as it currently stands. Therefore, we invite you to submit a revised version of the manuscript that addresses the points raised during the review process.

Please submit your revised manuscript by Jun 12 2026 11:59PM. If you will need more time than this to complete your revisions, please reply to this message or contact the journal office at plosone@plos.org. When you're ready to submit your revision, log on to https://www.editorialmanager.com/pone/ and select the 'Submissions Needing Revision' folder to locate your manuscript file.

- A letter that responds to each point raised by the academic editor and reviewer(s). You should upload this letter as a separate file labeled 'Response to Reviewers'.
- A marked-up copy of your manuscript that highlights changes made to the original version. You should upload this as a separate file labeled 'Revised Manuscript with Track Changes'.
- An unmarked version of your revised paper without tracked changes. You should upload this as a separate file labeled 'Manuscript'.If you would like to make changes to your financial disclosure, please include your updated statement in your cover letter. Guidelines for resubmitting your figure files are available below the reviewer comments at the end of this letter.

If applicable, we recommend that you deposit your laboratory protocols in protocols.io to enhance the reproducibility of your results. Protocols.io assigns your protocol its own identifier (DOI) so that it can be cited independently in the future. For instructions see: https://journals.plos.org/plosone/s/submission-guidelines#loc-laboratory-protocols. Additionally, PLOS ONE offers an option for publishing peer-reviewed Lab Protocol articles, which describe protocols hosted on protocols.io. Read more information on sharing protocols at https://plos.org/protocols?utm_medium=editorial-email&utm_source=authorletters&utm_campaign=protocols.

As the corresponding author, your ORCID iD is verified in the submission system and will appear in the published article. PLOS supports the use of ORCID, and we encourage all coauthors to register for an ORCID iD and use it as well. Please encourage your coauthors to verify their ORCID iD within the submission system before final acceptance, as unverified ORCID iDs will not appear in the published article. *Only*  the individual author can complete the verification step; PLOS staff *cannot*  verify ORCID iDs on behalf of authors.

We look forward to receiving your revised manuscript.

Kind regards,

Ka Ming Chow

Academic Editor

PLOS One

Journal Requirements:

1. If the reviewer comments include a recommendation to cite specific previously published works, please review and evaluate these publications to determine whether they are relevant and should be cited. There is no requirement to cite these works unless the editor has indicated otherwise.

2. Please review your reference list to ensure that it is complete and correct. If you have cited papers that have been retracted, please include the rationale for doing so in the manuscript text, or remove these references and replace them with relevant current references. Any changes to the reference list should be mentioned in the rebuttal letter that accompanies your revised manuscript. If you need to cite a retracted article, indicate the article’s retracted status in the References list and also include a citation and full reference for the retraction notice.

**Additional Editor Comments:**

Thank you for the opportunity to review the resubmission of the manuscript. In general, the revised manuscript is concise and easy to follow the presentation. To further improve the quality of the manuscript, please find some comments as follows:

1. In Abstract (line 56, page 3), it is stated that "Motivational strategies (34.2%) or behavioural change theory (6.3%) to encourage physical activity participation were infrequently included". For the motivational strategies, I don't think 34.2% is regarded as infrequently included. Suggest not to interpreting it as "infrequent", but only report the figure.

2. In Abstract, the conclusions (line 64-65, page 3) stated that "five future research recommendations have been proposed". It is suggested illustrating these recommendations briefly to address the last objective of the review if word limit doesn't exceed.

3. In Results (line 300, page 14), "the total number of studies (n=79) will be used ...." Past tense should be used instead of future tense.

4. Some formatting issues should be corrected, including space between 50-60 years (line 368, page 39), 60 months (5 years) (line 664, page 56), twelve should be presented as 12 (line 689, page 57).

5. In line 725, page 58, "The use of CERT identified that only 34.2% ...." Suggest deleting the word "only".

6. In Conclusion, the fourth recommendations for future research "Identify effective psychological theories to enhance long-term PA participation in this population" (line 833, page 63). Why psychological theories should be identified, not behavioural change theories? Justification should be provided.

[Note: HTML markup is below. Please do not edit.]

Reviewers' comments:

Reviewer's Responses to Questions

**Comments to the Author**

Reviewer #2: All comments have been addressed

Reviewer #3: (No Response)

Reviewer #4: All comments have been addressed

**********

2. Is the manuscript technically sound, and do the data support the conclusions?

Reviewer #2: Yes

Reviewer #3: Yes

Reviewer #4: Yes

**********

3. Has the statistical analysis been performed appropriately and rigorously?

Reviewer #2: N/A

Reviewer #3: N/A

Reviewer #4: Yes

**********

4. Have the authors made all data underlying the findings in their manuscript fully available?

The PLOS Data policy

Reviewer #2: No

Reviewer #3: Yes

Reviewer #4: Yes

**********

5. Is the manuscript presented in an intelligible fashion and written in standard English?

Reviewer #2: Yes

Reviewer #3: Yes

Reviewer #4: Yes

**********

Reviewer #2: This is a well-written and comprehensive scoping review on an important topic; however, several areas need improvement to strengthen the paper. First, there is confusion between the terms “physical activity” and “exercise,” which are used interchangeably throughout the manuscript. The authors should clearly define both terms and use them consistently. Second, the justification for choosing a scoping review needs to be clearer, explaining why this approach is more suitable than a systematic review. The search strategy also requires better transparency by providing full details for all databases and clarifying how grey literature was searched.

In addition, the results are mainly descriptive, relying on percentages and counts, with limited deeper analysis. The paper would benefit from more interpretation and grouping of findings to provide meaningful insights. The discussion on behavioural change theory is also underdeveloped, despite being an important finding, and should be expanded. Similarly, outcome measures for lymphoedema vary widely across studies, but this is not fully analyzed or compared in a structured way.

The manuscript should also better address methodological reporting requirements. Please send the PRISMA checklist file at your earliest convenience. In addition, include a clear statement in the Methods section confirming compliance with PRISMA guidelines and provide registration details (if applicable). The limitations should be strengthened, particularly regarding language restriction (English only), lack of quality appraisal, and incomplete reporting of participant characteristics such as socioeconomic status and gender diversity.

Regarding the discussion section, while it is comprehensive and aligned with existing literature, it would benefit from clearer structuring. The authors are encouraged to more explicitly highlight key convergences, divergences, remaining gaps in the literature, and implications for clinical practice. This section is quite lengthy and could be improved by making it more concise and focused.

The conclusions are clear and generally consistent with the results, but they could be more practical by emphasizing specific actions for researchers and clinicians. Finally, the reference list is mostly relevant and up to date; however, a final check is recommended to ensure full compliance with the required referencing style and journal standards. Addressing these points will significantly improve the clarity, quality, and overall impact of the manuscript.

Reviewer #3: Abstract

• I would suggest regarding the results section, you present the number of studies together with the percentages.

• Provide at summary of least one key recommendation in the conclusion section.

Introduction

• Check the transition (logical flow) from line 71 through to 74.

Methods

• With respect to “Joanna Brigg’s Institute (JBI) methodology” be specific “Joanna Brigg’s Institute (JBI) methodology for conducting scoping reviews”

Search

• I would suggest making it “Search Strategy”

Study Selection

• What do you mean by “randomly selected articles?”

• Did you include the three papers that had missing data or further data was required? If yes, it would be important to provide a rationale why these studies were included in the review and whether the missing data had an impact on the overall outcome of the review.

Table 2

• For the randomized controlled trial studies, it would be important to communicate to readers which group served as the control or intervention.

• The authors, you indicated just a name for all the studies. Does that mean each study was conducted by just one researcher? If that is not the case, I would suggest you revise it.

Discussion

• The statement “clinical practice guidelines now recommend that PA is safe for this population” (line 337-338) needs to be referenced.

• Line 340, were all the studies included interventional studies?

• I would suggest you refine Line 358.

• Why do you think the time for inculcating physical activity in the treatment of breast cancer varied? What has previous literature found?

Conclusions

I would suggest having different headings for conclusions and recommendations

Reviewer #4: A well done and written scoping review, capturing a rather low-researched area of Oncology care

To improve:

Line 89 (hanging sentence): With the fear of exacerbating swelling often cited as a barrier to PA (14).

Line 102 and 103 (poor punctuation): PA is commonly defined as‘any bodily movement produced by skeletal muscles that results in energy expenditure’ (22), however, the term alone has been challenged for…

**********

what does this mean?). If published, this will include your full peer review and any attached files.

If you choose “no”, your identity will remain anonymous but your review may still be made public.

**Do you want your identity to be public for this peer review?**  For information about this choice, including consent withdrawal, please see our Privacy Policy

Reviewer #2: **Yes:** Ahmed Yahya Ayoub

Reviewer #3: No

Reviewer #4: **Yes:** Emmanuel Ejembi Anyebe

**********

[NOTE: If reviewer comments were submitted as an attachment file, they will be attached to this email and accessible via the submission site. Please log into your account, locate the manuscript record, and check for the action link "View Attachments". If this link does not appear, there are no attachment files.]

To ensure your figures meet our technical requirements, please review our figure guidelines: https://journals.plos.org/plosone/s/figures

You may also use PLOS’s free figure tool, NAAS, to help you prepare publication quality figures: https://journals.plos.org/plosone/s/figures#loc-tools-for-figure-preparation.

NAAS will assess whether your figures meet our technical requirements by comparing each figure against our figure specifications.

# Author comment: Author response to Decision Letter 2

**DOI:** 10.1371/journal.pone.0355189.r004

12 May 2026

Dear Academic Editor, Thank you for taking the time to read and respond to the submitted manuscript. We welcome your feedback and consequently have made amendments to the abstract by including a brief summary of the 5 recommendations for future research. This includes changing the wording from psychological theories to behavioural change theories. We have also changed the formatting, use of the word only/infrequent and the tense from present to past as suggested. Further details can be found in the Response to Reviewers document. Thank you.

Dear Reviewer 2, Thank you for taking the time to read and provide welcomed feedback to the submitted manuscript. We have addressed all your comments in the Response to Reviewers document. In summary we have included definitions for physical activity and exercise and justification for undertaking a scoping review. We have also expanded the data provided for the search strategy and several documents including the PRISMA checklist can be found in the supplementary data included. We have restructured the results, discussion, conclusions and strengths/limitations sections and feel that by addressing these as suggested it has strengthened the overall manuscript. Thank you.

Dear Reviewer 3, Thank you for your time and feedback in reviewing this manuscript. We have reviewed all your comments and addressed them in the Response to Reviewers document. In summary we have amended the abstract to include the numbers and percentages and added in a summary of the recommendations. We have also taken into consideration your recommendations to strengthen the introduction, methods, results, discussion and conclusions section and feel that this has improved the manuscript. Thank you.

Dear Reviewer 4, Thank you for taking the time to read and review the manuscript. We welcomed your feedback and have addressed all comments in the Response to Reviewers document. Thank you.

Attachment Submitted filename: Response_to_Reviewers_auresp_2.docx

# Peer review: Decision Letter 2

**DOI:** 10.1371/journal.pone.0355189.r005

**Authors:** Ka Ming Chow

19 Jul 2026

Core components of physical activity interventions in the prevention and management of breast cancer-related lymphoedema (BCRL): a scoping review

PONE-D-25-60618R2

Dear Dr. Henry,

We’re pleased to inform you that your manuscript has been judged scientifically suitable for publication and will be formally accepted for publication once it meets all outstanding technical requirements.

Within one week, you’ll receive an e-mail detailing the required amendments. When these have been addressed, you’ll receive a formal acceptance letter and your manuscript will be scheduled for publication.

An invoice will be generated when your article is formally accepted. Please note, if your institution has a publishing partnership with PLOS and your article meets the relevant criteria, all or part of your publication costs will be covered. Please make sure your user information is up-to-date by logging into Editorial Manager at Editorial Manager® and clicking the ‘Update My Information' link at the top of the page. For questions related to billing, please contact billing support.

If your institution or institutions have a press office, please notify them about your upcoming paper to help maximize its impact. If they’ll be preparing press materials, please inform our press team as soon as possible -- no later than 48 hours after receiving the formal acceptance. Your manuscript will remain under strict press embargo until 2 pm Eastern Time on the date of publication. For more information, please contact onepress@plos.org.

Kind regards,

Ka Ming Chow

Academic Editor

PLOS One

Reviewers' comments:

Reviewer's Responses to Questions

**Comments to the Author**

Reviewer #2: All comments have been addressed

Reviewer #3: All comments have been addressed

**********

2. Is the manuscript technically sound, and do the data support the conclusions?

Reviewer #2: Yes

Reviewer #3: Yes

**********

3. Has the statistical analysis been performed appropriately and rigorously?

Reviewer #2: N/A

Reviewer #3: N/A

**********

4. Have the authors made all data underlying the findings in their manuscript fully available?

The PLOS Data policy

Reviewer #2: Yes

Reviewer #3: Yes

**********

5. Is the manuscript presented in an intelligible fashion and written in standard English?

Reviewer #2: Yes

Reviewer #3: Yes

**********

Reviewer #2: The manuscript is accepted for publication in its current form. The study is well written and meets the journal requirements. No further comments or recommendations.

Reviewer #3: Thank you for responding to the comments. However, as I indicated in my previous comments, I suggest you indicate the group that served as control and intervention for the randomized control trial studies.

Thank you.

**********

what does this mean?). If published, this will include your full peer review and any attached files.

If you choose “no”, your identity will remain anonymous but your review may still be made public.

**Do you want your identity to be public for this peer review?**  For information about this choice, including consent withdrawal, please see our Privacy Policy

Reviewer #2: No

Reviewer #3: No

**********

# Editor report: Acceptance letter

**DOI:** 10.1371/journal.pone.0355189.r006

**Authors:** Ka Ming Chow

PONE-D-25-60618R2

PLOS One

Dear Dr. Henry,

I'm pleased to inform you that your manuscript has been deemed suitable for publication in PLOS One. Congratulations! Your manuscript is now being handed over to our production team.

At this stage, our production department will prepare your paper for publication. This includes ensuring the following:

* All references, tables, and figures are properly cited

* All relevant supporting information is included in the manuscript submission,

* There are no issues that prevent the paper from being properly typeset

You will receive further instructions from the production team, including instructions on how to review your proof when it is ready. Please keep in mind that we are working through a large volume of accepted articles, so please give us a few days to review your paper and let you know the next and final steps.

Lastly, if your institution or institutions have a press office, please let them know about your upcoming paper now to help maximize its impact. If they'll be preparing press materials, please inform our press team within the next 48 hours. Your manuscript will remain under strict press embargo until 2 pm Eastern Time on the date of publication. For more information, please contact onepress@plos.org.

You will receive an invoice from PLOS for your publication fee after your manuscript has reached the completed accept phase. If you receive an email requesting payment before acceptance or for any other service, this may be a phishing scheme. Learn how to identify phishing emails and protect your accounts at https://explore.plos.org/phishing.

If we can help with anything else, please email us at customercare@plos.org.

Thank you for submitting your work to PLOS One and supporting open access.

Kind regards,

PLOS ONE Editorial Office Staff

on behalf of

Dr. Ka Ming Chow

Academic Editor

PLOS One
